# Novel and potent anti-tumor and anti-metastatic di-2-pyridylketone thiosemicarbazones demonstrate marked differences in pharmacology between the first and second generation lead agents

**DOI:** 10.18632/oncotarget.6389

**Published:** 2015-11-25

**Authors:** Vit Sestak, Jan Stariat, Jolana Cermanova, Eliska Potuckova, Jaroslav Chladek, Jaroslav Roh, Jan Bures, Hana Jansova, Petr Prusa, Martin Sterba, Stanislav Micuda, Tomas Simunek, Danuta S. Kalinowski, Des R. Richardson, Petra Kovarikova

**Affiliations:** ^1^ Department of Pharmaceutical Chemistry and Drug Analysis, Faculty of Pharmacy in Hradec Kralove, Charles University in Prague, Heyrovskeho, Hradec Kralove, Czech Republic; ^2^ Department of Pharmacology, Faculty of Medicine in Hradec Kralove, Charles University in Prague, Simkova, Hradec Kralove, Czech Republic; ^3^ Department of Biochemistry, Faculty of Pharmacy in Hradec Kralove, Charles University in Prague, Heyrovskeho, Hradec Kralove, Czech Republic; ^4^ Department of Inorganic and Organic Chemistry, Faculty of Pharmacy in Hradec Kralove, Charles University in Prague, Heyrovskeho, Hradec Kralove, Czech Republic; ^5^ Molecular Pharmacology and Pathology Program, Department of Pathology and Bosch Institute, University of Sydney, Sydney, New South Wales, Australia

**Keywords:** Di(2-pyridyl)ketone 4, 4-dimethyl-3-thiosemicarbazone, di(2-pyridyl)ketone 4-cyclohexyl-4-methyl-3-thiosemicarbazone, anti-cancer agents, metabolism, pharmacokinetics

## Abstract

Di(2-pyridyl)ketone 4,4-dimethyl-3-thiosemicarbazone (Dp44mT) and di(2-pyridyl)ketone 4-cyclohexyl-4-methyl-3-thiosemicarbazone (DpC) are novel, highly potent and selective anti-tumor and anti-metastatic drugs. Despite their structural similarity, these agents differ in their efficacy and toxicity *in-vivo*. Considering this, a comparison of their pharmacokinetic and pharmaco/toxico-dynamic properties was conducted to reveal if these factors are involved in their differential activity. Both compounds were administered to Wistar rats intravenously (2 mg/kg) and their metabolism and disposition were studied using UHPLC-MS/MS. The cytotoxicity of both thiosemicarbazones and their metabolites was also examined using MCF-7, HL-60 and HCT116 tumor cells and 3T3 fibroblasts and H9c2 cardiac myoblasts. Their intracellular iron-binding ability was characterized by the Calcein-AM assay and their iron mobilization efficacy was evaluated. In contrast to DpC, Dp44mT undergoes rapid demethylation *in-vivo*, which may be related to its markedly faster elimination (T_1/2_ = 1.7 h for Dp44mT *vs*. 10.7 h for DpC) and lower exposure. Incubation of these compounds with cancer cells or cardiac myoblasts did not result in any significant metabolism *in-vitro*. The metabolism of Dp44mT *in-vivo* resulted in decreased anti-cancer activity and toxicity. In conclusion, marked differences in the pharmacology of Dp44mT and DpC were observed and highlight the favorable pharmacokinetics of DpC for cancer treatment.

## INTRODUCTION

Despite the substantial progress in anti-cancer therapy in the last two decades, cancer still remains a serious public health issue worldwide. Resistance to current chemotherapies, along with metastasis, are among the main complications that stifle effective clinical management of cancer. Hence, there is still an urgent need for novel, more efficient anti-tumor agents which can address these concerns.

**Figure 1:**
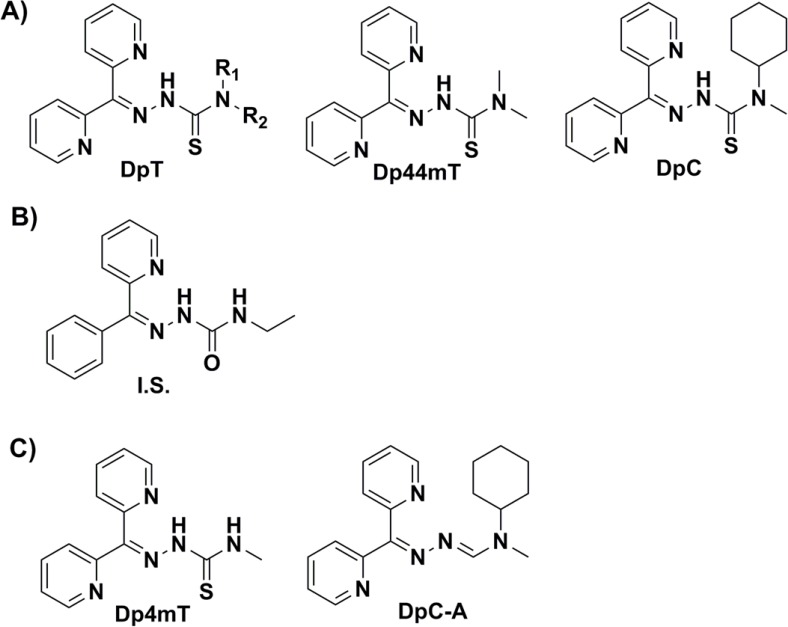
Line drawings of the chemical structures of the investigated compounds: A The general di(2-pyridyl)ketone thiosemicarbazone (DpT) structure and investigated thiosemicarbazones (di(2-pyridyl)ketone 4,4-dimethyl-3-thiosemicarbazone [Dp44mT] and di(2-pyridyl)ketone 4-cyclohexyl-4-methyl-3-thiosemicarbazone [DpC]); **B.** the internal standard (I.S.; (*Z*)-2-benzoylpyridine 4-ethylsemicarbazone); and **C.** the *in-vivo* metabolites of Dp44mT (di(2-pyridyl)ketone 4-methyl-3-thiosemicarbazone [Dp4mT]) and DpC (N-cyclohexyl-N′-(di(pyridin-2-yl)methylene)-N-methylformohydrazonamide; DpC-A) detected in rat plasma.

Ligands derived from the di(2-pyridyl)ketone thiosemicarbazone (DpT, Figure 1A) series are a promising group of novel anti-cancer drugs showing marked and significant benefits over the older thiosemicarbazone, Triapine^®^, which has been evaluated in clinical trials [1, 2]. Comprehensive chemical and pharmacological research on these new compounds has demonstrated their unique and selective efficacy and complex mechanism of activity [3, 4]. An important aspect of these novel thiosemicarbazones is their ability to inhibit both tumor growth and metastasis in a variety of tumor-types [1, 2, 5, 6].

These agents target cellular iron (Fe) and copper (Cu) as essential micronutrients and inhibit ribonucleotide reductase, which results in inhibition of DNA synthesis and cell growth [4, 7]. Furthermore, their complexes with Fe and Cu generate oxidative stress in cancer cells, which further augments their anti-proliferative activity [3, 5, 8] and this is also involved in their ability to overcome drug resistance [9]. These compounds have also been shown to target apoptotic and autophagic pathways [1, 3, 5, 10–12] and inhibit oncogenic signaling that regulates cancer cell growth, proliferation and metastasis [6, 13–15].

Based on its high efficacy and selectivity towards cancer cells, di(2-pyridyl)ketone 4,4-dimethyl-3-thiosemicarbazone (Dp44mT, Figure 1A) was selected as the first lead compound of the DpT group [1, 4]. However, cardiac fibrosis was demonstrated in mice after chronically repeated dosing at high non-optimal levels [1]. This observation initiated the development of the second generation of the DpT class, where di(2-pyridyl)ketone 4-cyclohexyl-4-methyl-3-thiosemicarbazone (DpC, Figure 1A) was identified as the new lead compound [2, 6].

While DpC is relatively similar to Dp44mT in terms of chemical structure (Figure 1A), it shows several advantages to the latter [2, 6]. These include: (1) DpC, unlike Dp44mT, does not induce cardiac fibrosis even when administered at markedly higher doses [2]; (2) Unlike Dp44mT, DpC does not induce oxidation of oxyhemoglobin to methemoglobin in red blood cells [16]; (3) DpC exhibits greater activity than Dp44mT *in-vivo*, even in poorly responding cancer-types, such as pancreatic cancer [6]; and (4) DpC showed marked and effective activity after both oral and intravenous administration [17], while Dp44mT is toxic after oral administration [17]. Moreover, the anti-proliferative efficacy of DpC *in-vivo* exceeds that of the current “gold standard” chemotherapeutic agent, gemcitabine, against a human pancreatic cancer xenograft [6]. Therefore, DpC is currently the most extensively investigated and active of the DpT class of ligands that is expected to enter clinical trials later in 2015.

Despite the prominent anti-proliferative activity of these novel thiosemicarbazones, there are still only scarce data regarding their metabolism and disposition. Although limited data on the *in-vitro* metabolism of DpC in human liver microsomal and S9 fractions have been reported [18], no data on the *in-vivo* pharmacokinetics (PK) of either of the lead compounds (Dp44mT and DpC, respectively) are available. This information may be particularly important to better understand the efficacy and safety of DpC *in-vivo* and to promote its further preclinical and clinical development [19]. Despite their similar chemical structures, both compounds may differ in their PK, which may explain, or at least contribute to, their distinct toxicity and efficacy profiles [19]. Given the fact that their mechanism of action involves redox reactions [3] and the compounds are sensitive to oxidation [20], formation of oxidative or other metabolites in cancer and/or cardiac cells cannot be excluded. Furthermore, the potential oxidative metabolites of the agents could have biological activity. The lack of the above mentioned data is largely caused by the relatively complicated analysis in biological materials, which is a prerequisite for such investigations. A major obstacle is the chelation of metals by these ligands within the chromatographic system and in biological materials, which results in significant difficulties in both sample preparation and LC-MS analysis.

Hence, the aim of this study was to investigate the pharmacological properties (metabolism and disposition) of the lead first and second generation DpT analogues (*i.e.*, Dp44mT and DpC) *in-vivo* using a new UHPLC-MS/MS method. Besides comparison of pharmacokinetic profiles of both compounds, their propensity towards *in-vivo* metabolism was studied and the pharmacodynamic and toxicodynamic effects of the detected and predicted metabolites were tested *in-vitro.*

## RESULTS

### Identification of the principal *in-vivo* metabolites of Dp44mT and DpC in plasma after administration to rats

The metabolism of both Dp44mT and DpC are crucial to understand in terms of their differential activity *in vivo* and facilitating the entrance of DpC into clinical trials [1–6]. Considering this, after administration of Dp44mT to rats, we found a significant amount of a metabolite (*m/z* 272) in the plasma (Supplemental Figure 1A). This was hypothesized to be a product due to *N*-demethylation, namely, Dp4mT (Figure 1C). The relatively higher retention of Dp4mT (relative retention of 1.17) as compared to Dp44mT was rather unusual and the reason for this observation remains unclear (Supplemental Figure 1A).

In contrast to Dp44mT, after administration of DpC, only a minor peak of a metabolite (*m/z* 322) was identified in the plasma (Supplemental Figure 1B). This molecule corresponded to the oxidative desulfuration of the thiourea structural moiety of DpC, resulting in *N*-cyclohexyl-*N′*-(di(pyridin-2-yl)methylene)-*N*-methylformohydrazonamide (DpC-A; Figure 1C). Notably, this metabolite was previously detected in our *in-vitro* metabolism study of DpC using human liver microsomes/S9 fractions [18].

### Investigation of the possible metabolic transformation of Dp44mT and DpC in cancer and cardiac cells *in-vitro*

Considering the results after the administration of Dp44mT and DpC to rats, studies then assessed their metabolism *in-vitro*. Only a few rather minor peaks of the putative metabolites were found after incubation of the thiosemicarbazones with either MCF-7 (Supplemental Figures 2 and 3) or H9c2 cells (data not shown). Besides DpC-A (*m/z* 322; Figure 1C) found in this study in rat plasma, we detected other products of oxidative desulfuration of the thiosemicarbazone moiety. These included: di(2-pyridyl)ketone 4-cyclohexyl-4-methylsemicarbazone (DpC-S; *m/z* 338, Supplemental Figure 4A) and di(2-pyridyl)ketone 4,4-dimethylsemicarbazone (Dp44mS; *m/z* 270; Supplemental Figure 4A). In addition, we observed minor cleavage of the hydrazone bond to liberate di(2-pyridyl)ketone (DpK; *m/z* 185, Supplemental Figure 4A). Importantly, all compounds detected in the cells incubated with the thiosemicarbazones were also found in the control (cell-free) media and PBS buffer incubated with Dp44mT and DpC at approximately the same ratio to the parent thiosemicarbazones (Supplemental Figures 2 and 3). This finding suggests that all these compounds were formed by slow chemical decomposition of the thiosemicarbazones during the incubation at 37°C and that the MCF-7 or H9c2 cell lines did not show any distinct metabolic contribution to this process.

### Development and validation of a UHPLC-MS/MS method for the pharmacokinetic experiments

Following the identification of the major *in-vivo* metabolites (Dp4mT for Dp44mT and DpC-A for DpC), the development and validation of a fast UHPLC-MS/MS assay of these compounds in plasma was performed. The UHPLC column was selected based on our previous experience [18] and the mobile phase composition was optimized to reach acceptable separation of all compounds during a reasonable run time (up to 10 min). Utilization of EDTA in several steps of the analysis was recognized as a key point in the reduction of metal complex formation from the ligands during chromatography to enable sufficient sensitivity, reproducibility and overall acceptable chromatography. Exposure of the MS instrument to EDTA was limited by employment of a switching valve.

**Figure 2:**
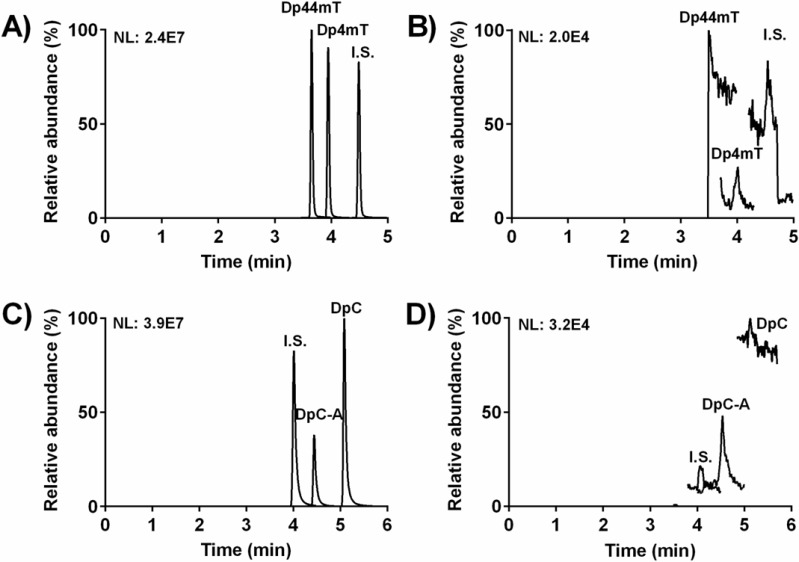
Representative chromatograms from UHPLC-MS/MS analyses: A Dp44mT, its metabolite, Dp4mT, and the I.S. in plasma at concentrations of 0.7 μmol·L^−1^ (Dp44mT and Dp4mT) and 0.25 μmol·L^−1^ (I.S.); **B.** the corresponding blank for **A.**; **C.** DpC, its metabolite, DpC-A, and the I.S. in plasma at concentrations of 1.5 μmol·L^−1^ (DpC and DpC-A) and 1 μmol·L^−1^ (I.S.); and **D.** the corresponding blank for **C.** The chromatograms were recorded in selected reaction monitoring and the intensity of the signal is presented as normalization level values (NL).

A structurally-related compound, (*Z*)-2-benzoylpyridine 4-ethylsemicarbazone, was found to be an optimal internal standard (I.S.; Figure 1B) for both Dp44mT and DpC. The representative chromatograms from the UHPLC-MS/MS analyses are illustrated in Figure 2. These include analysis of Dp44mT and its metabolite, Dp4mT, in plasma along with the I.S. (Figure 2A), relative to the corresponding blank (Figure 2B). In addition, a UHPLC-MS/MS chromatogram is shown that demonstrates DpC, DpC-A and the I.S. in plasma (Figure 2C) relative to the corresponding blank (Figure 2D).

Considering the sample preparation of rat plasma prior analysis, both protein precipitation (PP) and solid-phase extraction (SPE) provided only poor extraction recovery (≤ 44 and 40% for PP and SPE, respectively). Liquid-liquid extraction (LLE) to dichloromethane showed higher recovery (>50%), but it suffered from poor reproducibility. Finally, we developed a procedure combining PP and LLE that provided higher extraction efficiency and reproducibility over any other sample pre-treatment technique examined. We demonstrated that the presence of large amounts of EDTA, as well as the addition of another thiosemicarbazone (namely, Dp4eT; [5]) prior to protein precipitation, improved the reproducibility of the extraction.

The developed UHPLC-MS/MS assay met general validation criteria that corresponded to FDA guidelines [21]. Selectivity was confirmed with no significant interference from the matrix being detected (Figure 2B, 2D). Linearity, precision, accuracy, recovery and matrix effect of the methods are documented in Supplementary Tables 1-2. No significant decomposition of the compounds (*i.e.*, Dp44mT, Dp4mT, DpC and DpC-A) was observed either up to 7 days stored at −80°C or after 24 h storage in the autosampler at 10°C (Supplementary Table 3). A dilution integrity test proved acceptable precision and accuracy (Supplementary Table 3) [21].

### Pharmacokinetic experiments

**Figure 3:**
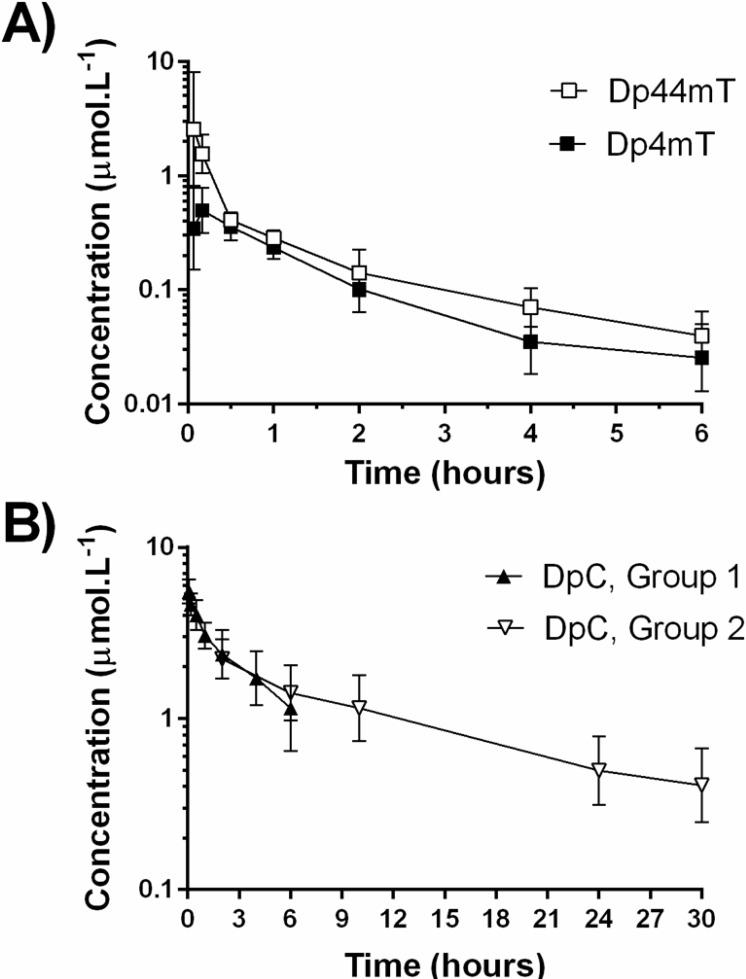
Geometric mean plasma concentration-time profiles of Dp44mT, Dp4mT and DpC: **A** Dp44mT and its metabolite, Dp4mT; and **B.** DpC in plasma after *i.v.* administration of the thiosemicarbazones to rats at a dose of 2 mg·kg^−1^ (7.0 μmol·kg^−1^ of Dp44mT and 5.66 μmol·kg^−1^ of DpC) as a slow bolus. Data are presented as mean ± S.D. (*n* ≥ 6). Dp44mT, Dp4mT and DpC, Group 1 – the blood was sampled from 0 to 6 h*;* DpC, Group 2 - the blood was sampled from 2 to 30 h; DpC.

The geometric mean (±S.D.) plasma concentration-time profiles of Dp44mT and its metabolite, Dp4mT, observed following a single intravenous administration of Dp44mT (2 mg·kg^−1^, *i.e.,* 7 μmol·kg^−1^) are shown in Figure 3A. Over the sampling interval of 6 h, the decrease of the mean Dp44mT concentrations was apparently biphasic. Its value at 6 h was less than 2 % of the mean C_max_ (2.55 μmol·L^−1^) found at the first sampling interval (4 min).

The geometric mean plasma concentration-time profiles of DpC also given intravenously (2 mg·kg^−1^, *i.e.*, 5.66 μmol·kg^−1^) are shown separately for groups 1 and 2 in Figure 3B. Unlike Dp44mT, the concentration of DpC at 6 h was 21% of the maximum on the curve (Group 1), despite the fact that the study conditions were identical between the two agents. This observation demonstrated that despite their clear structural similarity (Figure 1A), the compounds showed markedly different pharmacokinetics *in-vivo*. The relatively slow decrease of the mean DpC concentrations required additional experiments to describe the elimination phase of the concentration-time profile. Considering this, sampling was then continued for up to 30 h (Group 2; Figure 3B).

### Non-compartmental pharmacokinetic analysis

The pharmacokinetic characteristics estimated using non-compartmental analysis (NCA) of the mean concentration profiles are listed in Table 1. The mean extrapolated AUC of both agents (8 and 19% for Dp44mT and DpC, respectively) confirmed that the blood sampling was sufficient for the analysis. Comparison of these data for both agents also indicated marked differences in their total AUC, clearance and half-lives of elimination. Comparison of C_max_ and AUC of the metabolite, Dp4mT, and parent compound (Dp44mT) confirmed that Dp4mT is an important metabolite. Furthermore, it is notable that the metabolite achieved its C_max_ soon after the administration of the drug (T_max_ = 10 min; Table 1).

**Table 1:** Pharmacokinetic parameters of Dp44mT, its metabolite, Dp4mT, and DpC determined by non-compartmental analysis

Compound	C_max_ (μmol·L^−1^)	T_max_ (min)	AUC_0-tlast_ (h·μmol·L^−1^)	T_last_ (h)	AUC (h·μmol·L^−1^)	CL (L·h^−1^·kg^−1^)	V_z_ (L·kg^−1^)	lamda_z_ (h^−1^)	t_1/2_ (h)
Dp44mT	2.55	4.0	1.34	6	1.46	4.78	15.0	0.318	2.2
Dp4mT	0.496	10	0.671	6	0.748	NA	NA	0.346	2.0
DpC	5.51	4.0	32.2	30	39.8	0.142	2.77	0.0515	13.5

The studied agents (Dp44mT and DpC) were administered *i.v.* at the dose of 2 mg·kg^−1^ (7.0 μmol·kg^−1^ of Dp44mT and 5.66 μmol·kg^−1^ of DpC) to rats (*n* = 6 and *n* = 14 for Dp44mT and DpC, respectively). The PK parameters of the agents and Dp4mT metabolite were estimated by non-compartmental analysis from geometric mean concentration *vs*. time curves.

Abbreviations: NA- not applicable; C_max_ – maximum concentration; T_max_ – time to maximum concentration, AUC_0-tlast_ - area under the plasma concentration–time curve from zero up to a time t; T_last_ – last sampling interval; AUC- area under the plasma concentration–time curve from zero up to infinity; CL- total plasma clearance; Vz - apparent volume of distribution; lamda_z_ - terminal rate constant of elimination; t_1/2_- elimination half-life.

For DpC, only a formohydrazonamide metabolite was detected in the plasma (DpC-A; Figure 1C), but only in very small amounts (well below the LLOQ of the method). Hence, the maximal estimated AUC of the formohydrazonamide metabolite was lower than 1% of that of the parent drug, which suggests it is of minor importance.

### Population pharmacokinetic analysis

**Figure 4:**
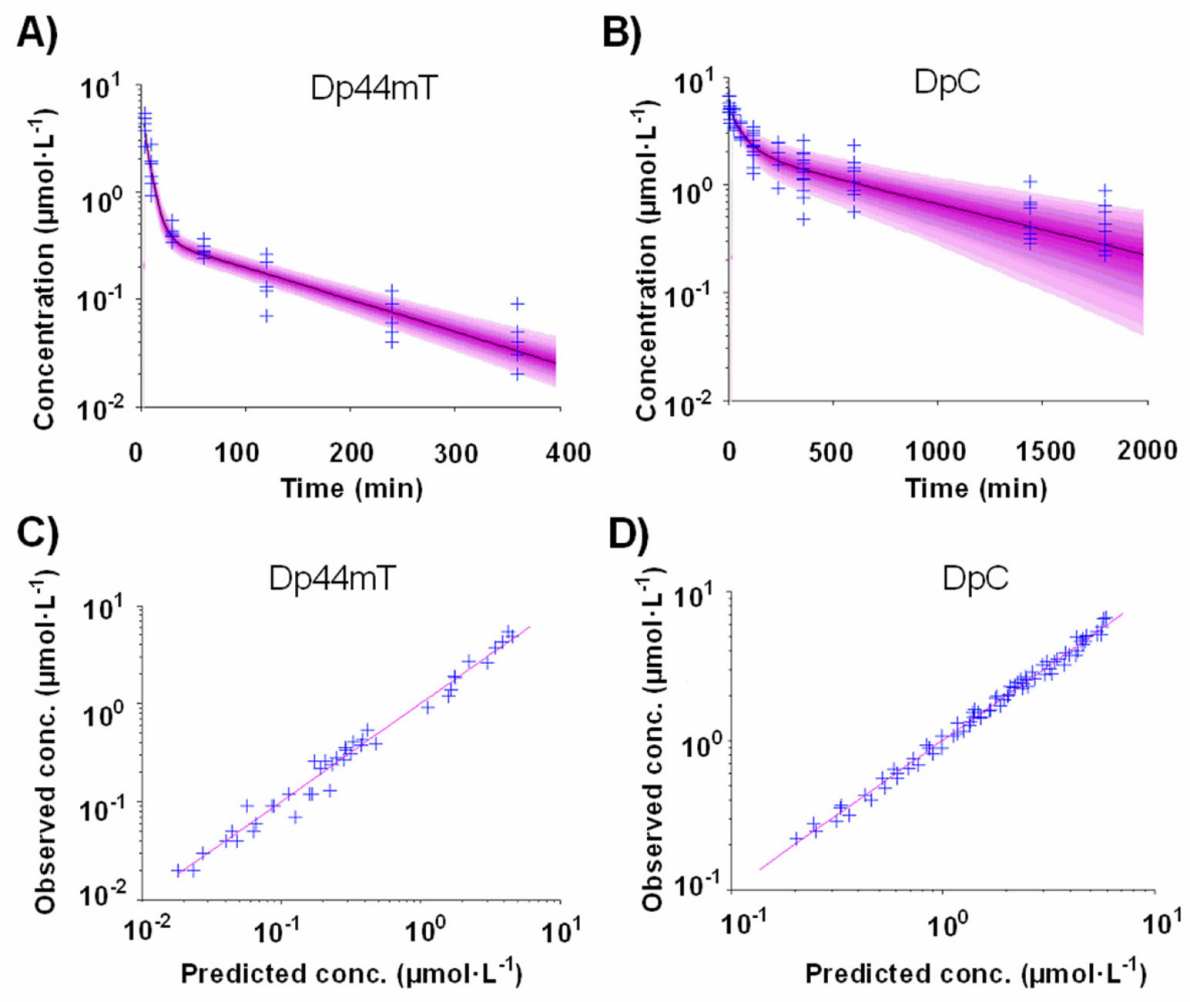
Predictive performance of the population models for A Dp44mT and **B.** DpC. The lines are the median predictions from the population models and the shaded areas are the 90% prediction intervals. The crosses show the observed concentrations. Scatter plot of the observed *vs.* individual predicted concentrations for **C.** Dp44mT and **D.** DpC.

Statistical evaluation and graphical inspection of the goodness-of-fit plots indicated that the plasma concentrations of Dp44mT, as well as DpC, are well described by an open two-compartment model with first-order elimination from the central compartment (Figure 3). The goodness-of-fit plots demonstrate that the data were well described by the population models (Figure 4). The statistical summary of *post hoc* Bayesian estimates for individual pharmacokinetic parameters are given in Table 2. Individually predicted concentrations of both compounds agreed well with the observed values: the mean prediction error achieved was −2.4% (95% CI: −9.3-4.5) and −0.4% (95% CI: −2.1-1.4), for Dp44mT and DpC, respectively. The mean absolute prediction errors were 16.8% (95% CI: 12.6-21.1) and 6.9 % (95% CI: 6.0-7.8) for Dp44mT and DpC, respectively.

**Table 2:** Statistical summary of the individual Bayesian estimates for the pharmacokinetic parameters of Dp44mT and DpC

Compound	C_max_ (μmol·L^−1^)	T_max_ (min)	V_c_ (L·kg^−1^)	V_p_ (L·kg^−1^)	Q (L·h^−1^·kg^−1^)	CL (L·h^−1^·kg^−1^)	t_1/2alpha_ (min)	t_1/2beta_ (h)	AUC (h·μmol·L^−1^)
Dp44mT	3.78 ± 1.33	5.0 ± 2.4	1.02 ± 0.12	5.44 ± 1.70	5.11 ± 0.66	4.31 ± 0.66	4.3 ± 0.54	1.7 ± 0.30	1.66 ± 0.25
DpC	5.57 ± 0.89	4.0 ± 0.0	1.05 ± 0.08	1.19 ± 0.68^a^	0.50 ± 0.11^b^	0.186 ± 0.104^b^	40 ± 5.0^b^	10.7 ± 4.0^b^	37.2 ± 16.0

The studied agents were administered *i.v.* to rats (*n* = 6 and *n* = 14 for Dp44mT and DpC, respectively) at the dose of 2 mg·kg^−1^ (7.0 μmol·kg^−1^ of Dp44mT and 5.66 μmol·kg^−1^ of DpC). Data are presented as mean ± S.D. Observed C_max_ and T_max_ values are reported (in the case of DpC, the data were evaluated in Group 1 only).

Statistical significance (unpaired Student's *t*-test with Welch's correction): “a” *P*<0.01 and “b” *P*<0.001. Dose-corrected characteristics for Dp44mT vs. DpC, respectively: C_max_/dose = 0.54 ± 0.19 vs. 0.98 ± 0.16, *P*<0.01 and AUC/dose = 0.24 ± 0.036 vs. 6.57 ± 2.83, *P*<0.001.

Abbreviations: C_max_ – maximum concentration; T_max_ – time to a maximum concentration, V_p_ – volume of peripheral compartment; V_c_ – volume of central compartment; Q – intercompartmental clearance; CL - total clearance, t_1/2alpha_ – initial (distribution) half-life; t_1/2beta_ - elimination half-life. AUC - area under the plasma concentration–time curve from zero to infinity

Examining the Bayesian estimates for the pharmacological parameters of Dp44mT and DpC, the dose-corrected individual C_max_ was higher for DpC than Dp44mT (by 80%, *p*<0.01; Table 2). In contrast, the T_max_ values and volumes of the central compartment (V_c_) were similar. On the other hand, both compounds differed in the volume of their peripheral compartments (V_p_) and inter-compartmental (distribution) clearance (Q), with both indices being much higher for Dp44mT (Table 2). The latter parameter also corresponded with the markedly shorter t_1/2α_ observed for Dp44mT relative to DpC. However, Dp44mT also showed much lower dose-corrected total AUC (28-fold), higher clearance (23-fold) and markedly shorter (6.3-fold) terminal half-life of elimination (t_1/2β_), as compared to DpC (Table 2).

### Pharmacological and toxicological assessments of metabolites

### Anti-proliferative activities and toxicities of Dp44mT, DpC and their metabolites

Next, the effects of the parent compounds (Dp44mT and DpC) and their metabolites (Dp4mT and DpC-A) on the proliferation/viability of cancer cell lines (HL-60, MCF-7 and HCT116) and non-cancer cell lines (H9c2 and 3T3) were assessed. As metabolism of the thiosemicarbazones could be species-dependent, the same experiments were also done with other compounds detected in this study *in-vitro* (*i.e.,* DpK, Dp44mS and DpC-S), as well as those previously observed in incubations with a human microsome/S9 fraction [18]. These experiments provided additional information of pharmacological activity and toxicity of the metabolites that could occur in other animal species. Furthermore, these results may help to elucidate the differential toxicity and efficacy of Dp44mT relative to DpC [2, 6]. The results of these experiments are shown in the Supplementary Tables and Supplemental Figures. These studies were done to understand the potential biological activity of these metabolites, which could contribute to the overall efficacy or toxicity of the parent agents, Dp44mT or DpC.

**Figure 5:**
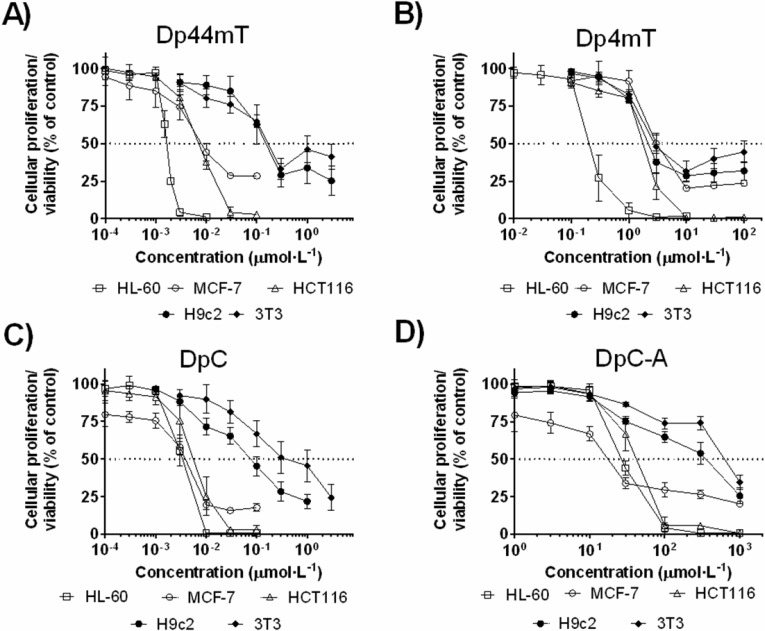
Cytotoxic effects of the parent thiosemicarbazones (Dp44mT and DpC) and their respective metabolites (Dp4mT and DpC-A): A Dp44mT; **B.** Dp4mT; **C.** DpC and **D.** DpC-A. Anti-proliferative and cytotoxicity effects of the compounds were studied at different concentrations after 72 h/37°C incubations with the cancer cell lines (HL-60, MCF-7 and HCT116) and non-cancer cell lines (H9c2 and 3T3). Proliferation/viability was determined using the MTT assay. Data are presented as mean ± S.D. (*n* ≥ 4).

Dp44mT was highly cytotoxic against all cancer cells examined, where the IC_50_ values ranged from 2 to 9 nmol·L^−1^ (Figure 5A and Table 3). In contrast, it showed only moderate cytotoxicity towards non-cancer H9c2 myoblasts (IC_50_ = 124 ± 49 nmol·L^−1^), as well as 3T3 fibroblasts (IC_50_ = 157 ± 51 nmol·L^−1^). The major metabolite of Dp44mT in rats, Dp4mT, showed significant activity only in the HL-60 cell line (IC_50_ = 0.250 ± 0.055 μmol·L^−1^), while in other cancer cells, the IC_50_ values ranged from 1.7 to 4.1 μmol·L^−1^ and this was comparable to its anti-proliferative activity in non-cancer cells (Figure 5B and Table 3). These results corresponded to previous studies demonstrating the markedly lower activity of Dp4mT relative to Dp44mT in a range of neoplastic cell lines [5].

**Table 3:** Cytotoxicity parameters of the studied thiosemicarbazones (Dp44mT, DpC) and their respective metabolites (Dp4mT and DpC-A) towards cancer (HL-60, MCF-7 and HCT116) and non-cancer (H9c2 and 3T3) cell lines

	IC_50_ (μmol·L^−1^)				
Compound	HL-60	MCF-7	HCT116	H9c2	3T3
Dp44mT	0.002 ± 0.000	0.009 ± 0.002	0.006 ± 0.001	0.124 ± 0.049	0.157 ± 0.051
Dp4mT	0.250 ± 0.055	4.106 ± 0.831	1.731 ± 0.160	3.250 ± 0.641	3.598 ± 0.664
DpC	0.003 ± 0.001	0.003 ± 0.001	0.005 ± 0.001	0.085 ± 0.013	0.412 ± 0.177
DpC-A	32.251 ± 3.854	19.527 ± 6.268	34.062 ± 6.349	257.203 ± 73.567	639.715 ± 65.874

The compounds were incubated with cells for 72 h/37°C. Cell proliferation/viability was determined using the MTT assay and the IC_50_ values (half-maximal inhibitory concentrations) were calculated using CalcuSyn 2.0 software. Data are presented as mean ± S.D. (*n* > 4).

DpC showed anti-cancer efficacy comparable with Dp44mT against cancer cells and decreased anti-proliferative activity against non-cancer cells (Table 3). The IC_50_ values of DpC against cancer cells ranged from 3 to 5 nmol·L^−1^, while its IC_50_ value against non-cancer cell lines was 85 and 412 nmol·L^−1^ for H9c2 and 3T3 cells, respectively (Figure 5C and Table 3). The formohydrazonamide metabolite (DpC-A) was relatively ineffective (IC_50_ ≥ 19 μmol·L^−1^) against all cancer cell lines and was also non-toxic (IC_50_ ≥ 257 μmol·L^−1^) towards non-cancer cells (Figure 5D and Table 3).

All other putative metabolites and products of chemical degradation in the medium of both compounds were found to be markedly less toxic against both cancer and non-cancer cells in comparison with the parent thiosemicarbazones (Supplemental Figure 5 and Supplementary Table 4).

### Intracellular labile iron chelation efficacy and the ability of Dp44mT, DpC and their metabolites to increase cellular iron mobilization and reduce iron uptake from transferrin

**Figure 6:**
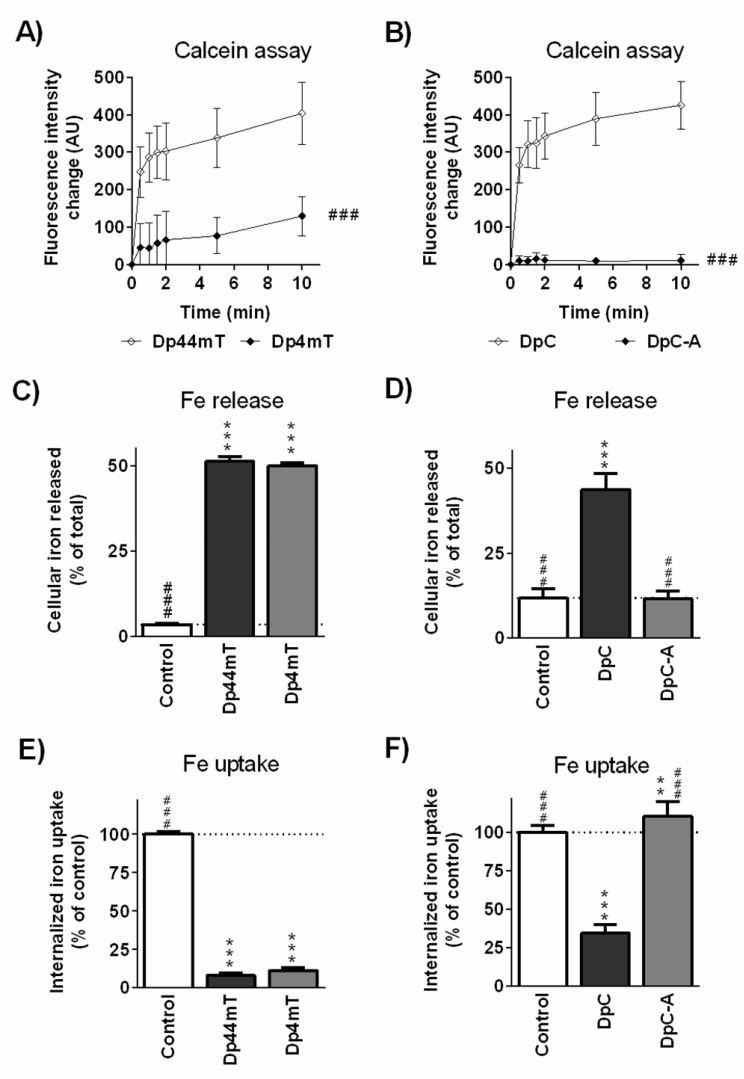
Iron chelation efficacy of the parent thiosemicarbazones, Dp44mT and DpC, and their respective metabolites, Dp4mT and DpC-A The ability of **A.** Dp44mT and its metabolite, Dp4mT, and **B.** DpC and its metabolite, DpC-A, to bind Fe from the cellular labile iron pools as determined by fluorimetric calcein-AM assay. (**C.**, **D.**) Mobilization of ^59^Fe from prelabeled MCF-7 cells by Dp44mT, DpC and their metabolites. (**E.**, **F.**) Inhibition of ^59^Fe uptake from ^59^Fe-transferrin by MCF-7 cells by Dp44mT, DpC and their metabolites. Statistical significance (ANOVA): # *p* < 0.05, ## *p* < 0.01, ### *p* < 0.001 compared to parent chelators (Dp44mT or DpC) and * *p* < 0.05, ** *p* < 0.01, *** *p* < 0.001 compared to the control (untreated) group. Data are presented as mean ± S.D. (*n* ≥ 4).

As iron chelation efficacy plays a significant role in the anti-proliferative activity of these and related ligands [2, 5, 22], studies were performed to assess their interaction with cellular iron pools that are essential for growth. The calcein-AM assay demonstrated the high intracellular chelation efficacy of Dp44mT (Figure 6A), while its main metabolite, Dp4mT, showed significantly lower relative efficacy (32% of Dp44mT chelation activity; Figure 6A). Notably, DpC showed intracellular chelation efficacy comparable to Dp44mT (Figure 6B), while its metabolite, DpC-A, showed only negligible chelation efficacy in this assay (2.5% of DpC chelation activity; Figure 6B). This is probably due to the loss of thiocarbonyl group in DpC-A (Figure 1C), which is a key site required for metal ion ligation [2].

Dp44mT also induced a significant (*p* < 0.001) increase in ^59^Fe mobilization from MCF-7 cancer cells prelabeled with ^59^Fe-transferrin (51% of cellular ^59^Fe; Figure 6C) relative to the control and similar results were also observed with its main metabolite, Dp4mT (Figure 6C). Notably, DpC was also very effective in this assay, mobilizing 44% of cellular ^59^Fe, while in contrast, its metabolite, DpC-A, was comparable (*p* > 0.05) to the control medium (Figure 6D). The ability of these agents to prevent ^59^Fe uptake from ^59^Fe-transferrin (Figure 6E, 6F) corresponded well with the results of the ^59^Fe mobilization studies. That is, the compounds with high activity at mobilizing ^59^Fe from MCF7 cells also had marked efficacy at preventing ^59^Fe uptake from ^59^Fe-transferrin by cells (Figure 6E, 6F).

Additional experiments with the other potential metabolites (*i.e.*, DpK, Dp44mS, DpC-S; Supplemental Figure 4A) using the calcein-AM assay showed their lower ability to bind intracellular Fe than their parent ligands, namely Dp44mT or DpC (Supplemental Figure 6A and 6B). The iron chelation efficacy of these predicted metabolites was then assessed using the more sensitive methods of assessing ^59^Fe mobilization or inhibition of ^59^Fe uptake in MCF-7 cells. These studies demonstrated that Dp44mS significantly (*p*<0.001) increased cellular ^59^Fe release relative to the control, being approximately half as effective as Dp44mT, while the activity of DpK was no greater than the control (Supplemental Figure 6C). On the other hand, the predicted DpC metabolite, DpC-S, demonstrated efficacy that was comparable to DpC, and markedly and significantly (*p*<0.001) increased ^59^Fe mobilization relative to the control (Supplemental Figure 6D).

In terms of the activity of these predicted metabolites at inhibiting internalized ^59^Fe uptake from ^59^Fe-transferrin, Dp44mS reduced ^59^Fe uptake to 64% of the control and was significantly (*p*<0.001) less effective than Dp44mT (Supplemental Figure 6E). In contrast, DpK did not have any significant (*p*>0.05) effect on ^59^Fe uptake (*p*>0.05) relative to the control (Supplemental Figure 6E). The activity of DpC-S at reducing ^59^Fe uptake from ^59^Fe-transferrin was marked and similar to DpC, resulting in a significant decrease (*p*<0.001) relative to the control (Supplemental Figure 6F).

## DISCUSSION

In this study, we found that the first and second generation ligands of the DpT series (namely, Dp44mT and DpC, respectively [2, 5, 6]) differ markedly in their metabolic profiles in rats *in-vivo*. Whereas Dp44mT was subjected to a pronounced single demethylation at the terminal N4 position, no such biotransformation occurred in the case of DpC. This suggests that the bulky cyclohexyl substitution of the tertiary nitrogen in DpC (Figure 1A) may sterically hinder the access of *N*-dealkylation enzymes (predominantly cytochrome P450 [23]) to this site. Hence, this structural difference leads to DpC being metabolically more stable. In fact, we detected only minor amounts of formamidrazone-like metabolite, indicating negligible oxidation of the thiocarbonyl moiety in rats *in-vivo*. A similar biotransformation pathway was previously reported for the related compound, 2-benzoylpyridine 4-ethyl-3-thiosemicarbazone (Bp4eT) in rats [24], where an amidrazone metabolite was identified in plasma. However, Bp4eT was markedly more prone to this metabolism pathway than DpC, with the AUC of the metabolite representing ≈ 20% of the parent drug [24].

In the current investigation, special attention was also paid to the search for products of hydrolysis of the hydrazone bond (namely, DpK), as this was previously shown to largely determine the short elimination half-life of the related aroylhydrazone chelators in rabbits (18-25 min) [25]. It is noteworthy that the present study confirmed that novel thiosemicarbazones did not suffer from the same problem. We identified only an insignificant amount of DpK in cells and cell media after *in-vitro* incubations with DpC or Dp44mT and were unable to detect any in the plasma taken in the PK experiments using rats. This is also in line with our previous work documenting the improved hydrolytic stability of different thiosemicarbazones over aroylhydrazone chelators in plasma and other biological materials *in-vitro* [26]. In addition, the markedly longer elimination half-lives observed for both DpC and Dp44mT suggest that significant plasma instability of these agents did not occur.

Our data also indicated that DpC or Dp44mT do not undergo significant metabolism in either cancer cells or heart-derived myoblasts. This largely excludes the possibility that these metabolites play a role in the pharmacological or toxicological effects ascribed to the parent compounds [1, 2, 5, 6]. While this class of DpT analogues can effectively redox cycle after chelating Fe or Cu in cells and this participates in their anti-cancer activity [3, 8], the lack of metabolites indicates that they are not significantly degraded by the redox stress that is induced. These findings also provide additional support to previous studies examining the cellular uptake of ^14^C-radiolabeled thiosemicarbazones and confirm that the total radioactivity measured in cancer cells is related to the parent compound and not its metabolites [27]. Notably, we also documented the slow chemical hydrolysis of Dp44mT and DpC in cell culture media and pharmacological evaluation of the hydrolytic products excluded their important role in the toxicity or efficacy of the parent compounds. Hence, both Dp44mT and DpC are highly stable compounds.

Identification of the principal metabolites *in-vivo* is essential for the subsequent targeted development and validation of quantitative assays for both thiosemicarbazones and their metabolites. We demonstrated that the low sensitivity and poor reproducibility initially encountered when designing these quantitative assays is likely due to the complexation of metal ions by these thiosemicarbazones during sample pre-treatment and/or within the chromatographic system. This property significantly complicated UHPLC-MS/MS assay of these agents in plasma. Significantly, our experiments revealed that the addition of another strong ligand (preferably EDTA) to all steps of analysis was beneficial for achieving reproducible results with sufficient sensitivity. Although the use of non-volatile salts mainly in the mobile phase is generally discouraged in MS-based assays, we did not observed any detrimental effects on the detector when a switching valve was employed, which is in line with the observations of others [28, 29].

The developed method allowed appropriate description of plasma concentration-time profiles after *i.v.* administration of both drugs to rats. Pharmacokinetic analyses of the data were performed using non-compartmental analysis and two-compartmental population analysis, both of which yielded very similar or complementary results. Unlike Dp44mT, two groups of animals were required to fully describe the PK profile of DpC due to the markedly prolonged elimination phase. The maximum concentrations of both compounds in the plasma were in the micromolar range with a slight (2-fold) difference in the dose-corrected C_max_. Importantly, these peak concentrations are nearly three orders of magnitude higher than their IC_50_ values determined *in-vitro* using different cancer cell lines in this investigation and previous studies [2]. This observation may, in part, explain the marked efficacy of these thiosemicarbazones against a range of different tumor-types *in-vivo* [1, 2, 5, 6].

As a consequence of the pronounced difference in total clearance, the characteristic of total exposure (AUC/dose) to DpC largely exceeded that of Dp44mT (>20-fold; Table 2), and importantly, almost identical data regarding the clearance of DpC were obtained with another rat strain (*i.e.*, Lewis rats, *n* = 8; data not shown). In contrast to DpC, Dp44mT was eliminated very effectively, as demonstrated by the total clearance (4.3-4.8 L^.^h^−1.^kg^−1^) approaching the sum of the liver and kidney blood perfusion rates in rats (3.3 and 2.2 L^.^h^−1.^kg^−1^, respectively [30]). The much higher clearance of Dp44mT relative to DpC may be at least partially explained by the rapid demethylation of the former agent.

The estimates for the volumes of distribution from the two-compartmental model provided evidence of rapid and extensive distribution of both drugs into tissues, which corresponds with their lipophilic nature and ability to easily and rapidly cross biological membranes [2, 27]. In fact, the initial (central) volumes of distribution exceeded both the volume of the circulation and also heavily perfused organs. Notably, there was a considerable difference in the volume of the peripheral compartment between Dp44mT and DpC (Table 2). However, the actual differences in tissue concentrations between these two compounds requires direct verification, as it depends on their overall partition coefficients between the plasma and other tissues (K_P_ = f_U,P_/f_U,T_) [31]. In the case of Dp44mT, a large fraction of the administered dose was eliminated before reaching pseudo-equilibrium, and unlike DpC, this resulted in the numerical value of V_z_ >> V_ss_ = (V_P_+V_T_) where V_z_ and V_ss_ represent the apparent volumes of distribution in terminal and steady state, respectively, and V_P_ and V_T_ are the volume of plasma and tissue, respectively.

Comparison of the PK parameters of both parent compounds in the present study to those of a related thiosemicarbazone, namely Bp4eT, previously reported in studies using rats [32], revealed several differences. In particular, Bp4eT showed an approximate 2-fold higher dose-corrected C_max_, but simultaneously slightly shorter elimination half-life (1.4 h) [32]. Hence, the elimination of both Dp44mT and Bp4eT was much faster than that of DpC, which may correspond with their higher propensity for metabolic elimination. The elimination of DpC may also differ from the clinically investigated thiosemicarbazone, Triapine^®^ [33]. In fact, while no preclinical data on the pharmacokinetics are available in the literature for this latter drug, the half-life of elimination after a single *i.v*. bolus was determined to be approximately 1 h in humans [33]. The only reference to its metabolism *in-vivo* describes the presence of hydroxylated or acetylated metabolites in the urine of Triapine^®^-treated patients [34]. Collectively, it can be suggested that even a relatively minor change in the chemical structure of thiosemicarbazones can have significant consequences on their pharmacokinetics. This observation indicates that it may be feasible to further optimize their PK properties *via* structural modifications to enhance efficacy.

Our *in-vitro* data showed little to no anti-proliferative activity of the demethylated Dp44mT metabolite, Dp4mT, *in-vitro* at the range of concentrations achievable *via* Dp44mT metabolism *in-vivo*. Markedly lower anti-cancer efficacy of this metabolite relative to the parent compound may correspond with the lower ability of Dp4mT to bind Fe from cellular pools, as documented by the calcein assay in the present study. On the contrary, we observed the similar capability of the metabolite and the parent drug to mobilize ^59^Fe from MCF-7 cells and prevent ^59^Fe uptake from ^59^Fe-transferrin, which corresponds with previous studies using SK-N-MC neuroepithelioma cells [5]. The discrepancy between these iron chelation assays may be explained by: (1) the limited drug exposure times in the calcein assay, which need not reflect the cumulative ability of the drug to mobilize ^59^Fe out of the cells after longer incubations; (2) the greater sensitivity of the radioisotope method using ^59^Fe relative to the calcein assay; and (3) the potentially different iron pools being estimated by the calcein and radioisotope methods. The significantly lower efficacy in intracellular chelation of Fe by Dp4mT relative to Dp44mT measured by the calcein assay better correlates with the decreased anti-proliferative activity of Dp4mT (Table 3). Of note, toxicity of the metabolite, Dp4mT, towards H9c2 myoblasts was an order of magnitude lower in comparison with Dp44mT, which suggests that Dp4mT is unlikely to be responsible for cardiotoxicity previously reported with Dp44mT after high, non-optimal doses [1].

The metabolism of DpC to the formohydrazonamide compound (DpC-A) led to significant attenuation of cytotoxic activity (Table 3), which is in line with the loss of Fe chelation efficacy observed in all Fe chelation assays (Figure 6). This is likely to be associated with the loss of the ligating sulfur atom, which is crucial in terms of Fe chelation [2]. Very low concentrations of this metabolite in plasma *in-vivo* (Supplemental Figure 1B), along with its negligible cytotoxicity (Table 3), strongly suggests that metabolism of DpC to DpC-A is unlikely to have any significant impact on the overall efficacy or safety of the drug.

In conclusion, this study demonstrates for the first time that the lead compounds of the first (Dp44mT) and second (DpC) generations of the DpT analogues differ considerably in their propensity towards biotransformation in rats. The rapid demethylation of Dp44mT to the metabolite, Dp4mT, may be involved in the markedly higher clearance, shorter half-life of elimination and smaller AUC in comparison to DpC. The metabolism of Dp44mT to Dp4mT resulted in a loss of anti-cancer activity, which may be associated with the lower chelation efficacy of Dp4mT measured by the calcein assay. The metabolite, Dp4mT, was relatively non-toxic to 3T3 fibroblasts and H9c2 cardiac myoblasts and did not appear to be responsible for cardiotoxicity observed at high, non-optimal doses of Dp44mT reported previously. The plasma concentrations of both Dp44mT and DpC markedly exceeded the effective IC_50_ values determined in cancer cells which could, in part, explain the significant activity of both agents *in-vivo*. However, the remarkably higher and longer exposure period found for DpC further highlights the *in-vivo* potential of this new lead compound. These findings may help to refine appropriate dosing schedules *in-vivo* and estimate PK/PD relationships. Moreover, these data can accelerate advanced preclinical development of DpC towards clinical evaluation and enable the targeted optimization of PK properties of newly developed thiosemicarbazones.

## MATERIALS AND METHODS

### Chemicals

The thiosemicarbazones, Dp44mT, DpC, di(2-pyridyl)ketone 4-ethyl-3-thiosemicarbazone (Dp4eT; Supplemental Figure 4B) and (*Z*)-2-benzoylpyridine 4-ethylsemicarbazone (internal standard; I.S., Figure 1B) were synthesized and characterized, as described previously [5, 6]. Other chemicals used for LC-MS analysis and sample preparation (all of HPLC, gradient or MS grade) as well as di(2-pyridyl)ketone (DpK, Supplemental Figure 4A; purity: 99%) were obtained from Sigma-Aldrich (Germany). Milli-Q water was prepared using a Millipore purification system (Merck-Millipore, Germany). Pooled blank rat plasma (with EDTA as an anti-coagulant) was obtained from healthy rats as described below. The ADS buffer (Millipore water (18.2 MΩ/cm) supplemented with 116 mM NaCl, 5.3 mM KCl, 1 mM CaCl_2_, 1.2 mM MgSO_4_, 1.13 mM NaH_2_PO_4_, 5 mM D-glucose, and 20 mM HEPES, pH 7.4) was prepared according to a previously published procedure [35].

### Synthesis of standards of putative metabolites

Di(2-pyridyl)ketone 4-methyl-3-thiosemicarbazone (Dp4mT, Figure 1C) was prepared according to a previously reported method [36]. Di(2-pyridyl)ketone 4,4-dimethylsemicarbazone (Dp44mS; Supplemental Figure 4A) was synthesized by the reaction of di(2-pyridyl)ketone (Sigma-Aldrich) and 4,4-dimethylsemicarbazone, as described in the Supplementary Materials and Methods. *N*-cyclohexyl-*N′*-(di(pyridin-2-yl)methylene)-*N*-methylformohydrazonamide (DpC-A, Figure 1C) was prepared by the oxidation of the parent drug, DpC, with hydrogen peroxide according to a procedure described previously [32]. Di(2-pyridyl)ketone 4-cyclohexyl-4-methylsemicarbazone (DpC-S; Supplemental Figure 4A) was prepared by the reaction of di(2-pyridyl)ketone (Sigma-Aldrich, Germany) with 4-cyclohexyl-4-methylsemicarbazide in ethanol.

The chemical structures described above and their purities were confirmed by ^1^H and ^13^C NMR and MS-ESI^+^. The details of these syntheses and the characterization of standards are described in the Supplementary Material.

### Cell culture

The MCF-7 human breast adenocarcinoma cell line was purchased from the European Collection of Cell Cultures (U.K.). The HL-60 human promyelocytic leukemia cell line, HCT116 human colorectal carcinoma cell line, H9c2 rat heart-derived myoblast cell line and 3T3 mouse embryo fibroblast line, were obtained from the American Type Culture Collection (VA, U.S.A.). Cells were cultured in Dulbecco's modified Eagle's medium (DMEM, Lonza, Switzerland) with or without (for MCF-7 cells only) phenol red, supplemented with 10% heat-inactivated fetal bovine serum (FBS; Lonza, Switzerland), 1% penicillin/streptomycin solution (Lonza, Switzerland) and 10 mM HEPES buffer (pH 7.0-7.6; Sigma-Aldrich, Germany). The HL-60 cell line was maintained in RPMI medium (Sigma-Aldrich, Germany) supplemented with 10% heat-inactivated FBS and 1% penicillin/streptomycin solution. All cell lines were cultured in 75 cm^2^ tissue culture flasks (Switzerland) at 37°C in a humidified atmosphere of 5% CO_2_. Sub-confluent cells or, in the case of HL-60 cells, a cell suspension, were sub-cultured every 3-4 days.

### Experimental animals

Male Wistar rats (230-310 g; *n* = 22) obtained from Velaz (Czech Republic) were kept in an air-conditioned room under a 12 h light-dark cycle, constant temperature and humidity and had free access to water and a standard laboratory pellet diet for rodents. All animal handling and procedures were approved and supervised by the Animal Welfare Body of the Faculty of Medicine in Hradec Kralove, Charles University in Prague. The investigation conformed to the Guide for the Care and Use of Laboratory Animals [37].

### *In-vivo* study of drug metabolism and disposition

The rats were anaesthetized with pentobarbital (30 mg^.^kg^−1^; *i.p.*) and then the *vena jugularis* and *arteria carotis* were prepared for drug administration and blood sample collection, respectively. Both Dp44mT and DpC were dissolved in a mixture of saline, PEG 300 and ethanol (5:4:1, *v/v/v*) and administered to rats at a dose of 2 mg^.^kg^−1^, as a slow *i.v*. bolus. Blood was then collected into EDTA-containing tubes, immediately centrifuged and the plasma kept at −80°C until being analyzed. The blood withdrawn from animals was compensated with an appropriate volume of saline. The animals were sacrificed by pentobarbital overdose.

Initially, for both compounds, rats (*n* = 2, in each group) were used for the *in-vivo* investigation of the thiosemicarbazone metabolites and also the preparation of the quantitative analytical method. For the full PK study, 6 rats were used for each compound (group 1) and the blood was sampled at 4, 10, 30, 60, 120, 240 and 360 min post-administration.

Due to the markedly slower elimination of DpC, an additional *in-vivo* study was performed to appropriately describe the elimination phase of this drug. The conscious rats (*n* = 8, group 2) were administered DpC (2 mg^.^kg^−1^) *via* slow *i.v.* bolus to the *vena saphena.* Blood was sampled from the retro-orbital plexus under light ether anesthesia during the following time intervals: 120, 360, 600, 1440 and 1800 min. Plasma samples were treated and analyzed using the analytical methods described below in the Section: *UHPLC-MS/MS methods for quantitative assay of the drugs and their metabolites.*

### Pharmacokinetic analysis

Standard non-compartmental analysis was performed implementing Kinetica software (version 4.0, Thermo Fisher Scientific Inc., MA, U.S.A.). Using naive data pooling, these data were combined into a geometric mean concentration-time curve from 6 animals per sampling interval (Dp44mT and its metabolite, Dp4mT) and from 6 to 14 animals per sampling interval (DpC). Maximum concentration (C_max_) and the time to maximum concentration (T_max_) were determined directly from the geometric mean *vs.* time profiles and from individual profiles. The area under the mean plasma concentration–time curve from zero up to the last sampling interval (AUC_0-tlast_) was calculated by a combination of the linear and log-linear trapezoidal methods. The area under the mean plasma concentration–time curve from zero up to infinity (AUC) was determined as the sum of the AUC_0–tlast_ and of the extrapolated part (*i.e.,* the ratio of the concentration predicted at the time interval of t_last_ and the terminal rate constant, λ_z_). The λ_z_ was estimated by linear regression of the log transformed concentrations in the terminal part of the curve.

Population pharmacokinetic modeling was performed using non-linear, mixed effect modeling as implemented in Monolix, version 4 (http://wfn.software.monolix.org). Pharmacokinetic parameters were estimated by computing the maximum likelihood estimator of the parameters without any approximation of the model (no linearization). This was performed using the Stochastic Approximation Expectation Maximization (SAEM) algorithm combined with a Markov chain Monte Carlo procedure. The between-animal variability in model parameters were ascribed to an exponential model and a proportional model was used to describe the residual variability. The goodness-of-fit was assessed by using several diagnostic plots (observed and predicted concentrations *vs.* time; observed concentrations *vs*. population predictions; weighted residuals *vs.* time; and weighted residuals *vs.* predictions). Individual parameter values were obtained as empirical Bayes estimates. Finally, the predicted individual concentrations were compared with the observed data using the mean prediction error and the mean absolute prediction error.

### *In-vitro* study of metabolism of drugs in breast cancer and cardiac myoblast cells

Breast cancer cells (MCF-7) and H9c2 heart-derived myoblasts were seeded in 100-mm Petri dishes (1,000,000 cells/dish) for 24 h/37°C. Freshly prepared stock solutions of Dp44mT and DpC in DMSO (Sigma-Aldrich, Germany) were diluted in culture medium to the required working concentration. The final concentrations of DMSO in these later solutions did not exceed 0.1%, which has been shown not to affect proliferation or cellular metabolism [22].

The cells were incubated for 12 h (37°C) with either Dp44mT or DpC at a concentration of 10 μmol·L^−1^. After this incubation, the cells were harvested, washed twice with ice-cold PBS buffer, centrifuged (790 x *g*) and precipitated with acetonitrile (200 μL/10^6^ cells) prior to the analysis. The overlaying medium was also analyzed to search for possible metabolites. In order to determine possible chemical decomposition, an identical protocol was performed with control medium and PBS buffer without cells. All samples were analyzed using HPLC-MS as described below in the Section: *HPLC-MS method to identify metabolites of the drugs.*

### HPLC-MS method to identify metabolites of the drugs

To search for the metabolites, plasma was precipitated with methanol or acetonitrile (plasma/solvent ratio – 1:3, *v/v*), treated using solid phase extraction (SPE; Discovery DSC-PH, Supelco, PA, U.S.A.), or liquid-liquid extraction with various solvents (ethyl acetate, toluene, dichloromethane) [24]. The cells and media were either precipitated or diluted with acetonitrile. These samples were then analyzed using HPLC-MS with ion trap mass analyzer implementing the settings described previously with minor modifications [24] (for details of the analyses see Supplementary Material, part 2.1). Importantly, particular attention was paid to the metabolites predicted previously from our *in-vitro* study [18]. The chemical structures of metabolites were suggested based on MS^n^ experiments and subsequently confirmed using the HPLC-MS analysis of the chemical standards synthesized for this purpose.

### UHPLC-MS/MS methods for quantitative assay of the drugs and their metabolites

#### Stock solutions, working solutions and quality control samples

Stock solutions (0.5 mg·mL^−1^) of all analytes (Dp44mT, Dp4mT, DpC and DpC-A), internal standard (I.S.) and auxiliary chelator (Dp4eT) were prepared by dissolving the appropriate amount of each agent in acetonitrile. Working solutions were then prepared by gradual dilution of the stock solutions with 50% acetonitrile until the desired concentrations were reached. Quality control samples were prepared by addition of the appropriate amount of working solutions to drug-free plasma.

#### Sample preparation

Plasma samples were treated with a combined protein precipitation and liquid-liquid extraction procedure according to the following protocol. One microliter of I.S. working solution (concentration of either 25 or 100 μmol·L^−1^ for Dp44mT or DpC, respectively) and 1 μL of Dp4eT solution (100 μmol·L^−1^) were added to the plasma samples (100 μL). The samples were then diluted with 100 μL of 2% aqueous solution of K_2_EDTA and mixed thoroughly. Thereafter, 200 μL of 1.25% NH_4_OH in acetonitrile followed by 800 μL of dichloromethane were added. This mixture was vortexed (5 min) and then centrifuged (10 min; 16,800 × *g*; 20°C). The organic layer was subsequently dried under a gentle flow of nitrogen. The residuum was reconstituted in either 50 μL or 100 μL of a K_2_EDTA solution (250 μmol·L^−1^) in 50% aqueous acetonitrile for Dp44mT or DpC, respectively, and analyzed.

#### Chromatographic conditions and MS settings

A Nexera UHPLC system coupled with LCMS-8030 triple quadrupole mass detector (both from Shimadzu, Japan) was used operating in ESI positive mode. The acquired data were processed using LabSolutions software (v. 5.60 SP2, 2013, Shimadzu, Japan). All separations were achieved on a column (Acquity UPLC^®^ BEH C18 1.7 μm, 2.1 × 50 mm, Waters, Ireland) protected with the same type of guard column. The column was flushed with 2 mmol·L^−1^ EDTA solution with acetonitrile (90:10, *v/v*) prior to the first use. The mobile phase consisted of 2 mmol·L^−1^ ammonium formate aqueous solution with the addition of K_2_EDTA (5 μmol·L^−1^, component A) and acetonitrile (component B) in gradient mode (see Supplementary Material, part 2.2). A switching valve was used and the mobile phase was allowed to enter the MS instrument merely for the time necessary for ESI stabilization and compound detection. The following conditions were used: a flow rate of 0.3 mL·min^−1^; an autosampler temperature of 10°C; a column temperature of 30°C; and injection volume of 4 μL. Prior to each analytical run, a mixture of 4.5 mmol·L^−1^ or 3.5 mmol·L^−1^of K_2_EDTA in the mobile phase of Dp44mT or DpC, respectively, was injected and eluted to waste. Quantitation was performed in selected reaction monitoring mode (SRM) using low resolution. The mass spectrometer set-up and detailed SRM parameters are specified in the Supplementary Materials and Methods (part 2.2).

#### Method validation

The analytical methods were validated according to the United States Food and Drug Administration Guidelines (Bioanalytical Method Validation) [21] with respect to selectivity, linearity, precision, accuracy, stability, recovery, matrix effects and dilution integrity. The following concentration ranges were used: 0.035-2 μmol·L^−1^ (Dp44mT and Dp4mT) and 0.150-3 μmol·L^−1^ (DpC and DpC-A). Details on validation procedures are specified in the Supplementary Material.

#### Characterization of pharmacological properties of the drugs and their metabolites *in-vitro*

Pharmacological properties of the following agents were assessed: (1) the parent thiosemicarbazones (Dp44mT and DpC); (2) their metabolites detected in this study in plasma (Dp4mT, DpC-A); (3) their putative metabolites (*i.e.* DpK, Dp44mS and DpC-S) identified from *in-vitro* studies in cell culture media and cancer cells; and (4) metabolites of these thiosemicarbazones identified from our previous studies *in-vitro* [18].

#### Proliferation and cytotoxicity studies

Cells were seeded in 96-well plates for 24 h/37°C at a density of 5,000 (MCF-7), 10,000 (HL-60), 2,000 (HCT116) and 10,000 (3T3 and H9c2) cells/well. For 3T3 and H9c2 cells, the medium was changed to serum- and pyruvate-free DMEM 24 h prior to the experiments. Anti-proliferative and cytotoxicity effects of the compounds were studied at different concentrations after incubations for 72 h/37°C. Viabilities of cells were determined using MTT assays (Sigma-Aldrich, Germany) according to the manufacturer's instructions on a Tecan Infinite 200M plate reader (Tecan, Austria). The proliferation/viability of treated groups was expressed as a percentage of the untreated controls (100%). The half maximal inhibitory concentrations resulting in a 50% reduction of cellular proliferation/viability (IC_50_) as compared to the untreated control value, were calculated using CalcuSyn 2.0 software (Biosoft, U.K.).

#### Iron chelation efficacy, mobilization of ^59^Fe from cells and ^59^Fe uptake from ^59^Fe-transferrin

Iron chelation efficiency of the compounds at a concentration of 10 μmol·L^−1^ was determined *via* 3 methods: (1) the calcein assay; (2) the ability of the ligands to induce ^59^Fe mobilization from cells prelabeled with ^59^Fe-transferrin; and (3) the efficacy of these agents to inhibit the uptake of ^59^Fe from ^59^Fe-transferrin [22, 38–40].

The fluorimetric calcein-AM assay was performed by established methods, as described previously [38, 39]. The Fe chelation efficiency of the metabolites in cells was expressed as a percentage of that of the parent chelator, Dp44mT or DpC (100%). For studies assessing the ability of these agents to induce ^59^Fe mobilization and inhibit ^59^Fe uptake from ^59^Fe-transferrin by cells, human transferrin (Sigma-Aldrich, Germany) was labeled with ^59^Fe (PerkinElmer, MA, USA) to generate ^59^Fe-transferrin at a final specific activity of 500 pCi/pmol Fe, as previously described [22, 39, 40]. The unbound ^59^Fe was removed by exhaustive vacuum dialysis against a large excess of 0.15 M NaCl buffered to pH 7.4 with 1.4% NaHCO_3_ by standard methods [22, 40].

To examine the ability of the studied substances to mobilize ^59^Fe from MCF-7 cells or inhibit ^59^Fe uptake from ^59^Fe-transferrin, established techniques were utilized [22, 40]. For ^59^Fe mobilization studies, cells were preincubated with ^59^Fe-transferrin (0.75 μmol·L^−1^) for 3 h/37°C, washed 4 times at 4°C on ice, and then reincubated with control medium or control medium containing the compounds (25 μmol·L^−1^) for 3 h/37°C. The release of ^59^Fe from the cells into the overlying medium was then examined by collecting both the cells and overlying medium and assessing ^59^Fe levels using a γ-counter. For studies examining the ability of the agents to inhibit ^59^Fe uptake from ^59^Fe-transferrin, cells were incubated for 3 h/37°C with ^59^Fe-transferrin (0.75 μmol·L^−1^) in the presence or absence of the agents (25 μmol·L^−1^), and then washed 4 times at 4°C on ice. The internalization of ^59^Fe by cells was then assessed by incubation for 30 min/4°C with the general protease, Pronase (1 mg/mL), utilizing standard techniques [22, 40]. The amount of internalized ^59^Fe in the presence of the agents was expressed as a percentage of ^59^Fe internalized by control cells incubated in the presence of control media alone.

#### Statistical analyses

Statistical analyses were performed using SigmaStat for Windows 3.5 (SPSS, CA, U.S.A.) and GraphPad Prism software, version 5.0 (GraphPad Software, CA, U.S.A). Data were expressed as the mean ± S.D. unless stated otherwise. Statistical significance was determined using Student's *t*-test or one-way ANOVA with a Bonferroni *post-hoc* test (comparisons of multiple groups against the corresponding control). The results were considered to be statistically significant when *p* < 0.05.

## SUPPLEMENTARY MATERIAL FIGURES AND TABLES



## Abbreviations

Dp44mS: di(2-pyridyl)ketone 4,4-dimethylsemicarbazone
Dp44mT: di(2-pyridyl)ketone 4,4-dimethyl-3-thiosemicarbazone
Dp4eT: di(2-pyridyl)ketone 4-ethyl-3-thiosemicarbazone
Dp4mT: di(2-pyridyl)ketone 4-methyl-3-thiosemicarbazone
DpC: di(2-pyridyl)ketone 4-cyclohexyl-4-methyl-3-thiosemicarbazone
DpC-A: *N*-cyclohexyl-*N′*-(di(pyridin-2-yl)methylene)-*N*-methylformohydrazonamide
DpC-S: di(2-pyridyl)ketone 4-cyclohexyl-4-methylsemicarbazone
DpK: di(2-pyridyl)ketone
DpT: di(2-pyridyl)ketone thiosemicarbazone
EDTA: ethylenediaminetetraacetic acid
ESI: electrospray ionization
IC_50_: half maximal inhibitory concentration
I.S.: internal standard
K_2_EDTA: ethylenediaminetetraacetic acid dipotassium salt
LLE: liquid-liquid extraction
PP: protein precipitation
SPE: solid-phase extraction
SRM: selected reaction monitoring
