# Supplementary material for: Novel and potent anti-tumor and anti-metastatic di-2-pyridylketone thiosemicarbazones demonstrate marked differences in pharmacology between the first and second generation lead agents
Source: Oncotarget. 2015 Nov 25;6(40):42411–28. doi: 10.18632/oncotarget.6389 (PMC4767442; doi:10.18632/oncotarget.6389)
Supplement: Supplementary file 1 [file oncotarget-06-42411-s001.pdf]

# Novel and potent anti-tumor and anti-metastatic di-2-pyridylketone thiosemicarbazones demonstrate marked differences in pharmacology between the first and second generation lead agents

**Supplementary Material**

## 1. Synthesis and the characterization of standards of the metabolites and their precursors

### *1.1. Di(2-pyridyl)ketone 4-methyl-3-thiosemicarbazone (Dp4mT)*

**Synthesis:** Di(2-pyridyl)ketone 4-methyl-3-thiosemicarbazone was prepared by the condensation of di(2-pyridyl)ketone (1.853 g, 0.010 mol) with 4-methyl-3-thiosemicarbazide (1.051 g, 0.010 mol; both from Sigma Aldrich, Munich, Germany) according to our previously described procedure (*Richardson, D. R. J. Med. Chem. 2006, 49, 6510-6521). **Yield:*** 63% (1.707 g) as brownish solid. **Characterization:** $^{1}H$ NMR (300 MHz, Acetone) δ 13.75 (s, 1H, NH), 8.82 (d, *J* = 4.9 Hz, 1H, Py), 8.57 (d, *J* = 4.8 Hz, 1H, Py), 8.52 (s, 1H, NH), 8.04 – 7.82 (m, 3H, Py), 7.64 – 7.57 (m, 1H, Py), 7.58 – 7.50 (m, 1H, Py), 7.47 – 7.38 (m, 1H, Py), 3.16 (d, *J* = 4.8 Hz, 3H, CH3). $^{13}C$ NMR (75 MHz, Acetone) δ 180.27, 156.84, 152.73, 149.20, 148.72, 141.12, 137.91, 137.62, 128.00, 125.18, 124.62, 124.35, 31.40. **MS-ESI$^{+}$:** $[M+H]^{+}$ 272.

### *1.2. Di(2-pyridyl)ketone 4,4-dimethylsemicarbazone (Dp44mS)*

**Synthesis:** Dimethylcarbamoyl chloride (1 mL, 1.17 g, 0.011 mol) was added dropwise to a stirred solution of hydrazine hydrate (1.087 g, 0.022 mol) in ethanol (30 mL). The resulting solution was stirred for 2 h and left in the refrigerator overnight. The precipitate was removed by filtration and the solution was concentrated and dried under high vacuum. The residue was dissolved in ethanol (30 mL) and di(2-pyridyl)ketone (0.88 g, 0.0048 mol) and 5 drops of glacial acetic acid were then added. The reaction mixture was refluxed for 1 h, cooled to room

temperature and evaporated. The product was obtained by silica gel column chromatography (mobile phase: ethyl acetate:acetone, 1:1, *v/v*). **Yield:** 39% (0.5 g) as a yellowish solid. **Characterization:** $^1$H NMR (500 MHz, DMSO) δ 8.82 – 8.79 (m, 1H, Py), 8.59 – 8.56 (m, 1H, Py), 7.98 – 7.91 (m, 2H, Py), 7.81 – 7.77 (m, 1H, Py), 7.55 – 7.51 (m, 1H, Py), 7.51 – 7.47 (m, 1H, Py), 7.46 – 7.41 (m, 1H, Py), 2.98 (s, 6H, $CH_3$). $^{13}$C NMR (126 MHz, DMSO) δ 156.35, 154.36, 151.94, 148.38, 147.72, 141.23, 137.77, 137.27, 126.38, 124.23, 123.82, 123.50, 35.99. **MS-ESI$^+$:** $[M+H]^+$ 270.

### *1.3. N-cyclohexyl-N'-(di(pyridin-2-yl)methylene)-N-methylformohydrazonamide (DpC-A)*

**Synthesis:** Hydrogen peroxide (30%; 0.3 mL) was added to a solution of DpC (75 mg, 0.21 mmol) in acetonitrile (7 mL). The reaction mixture was stirred at room temperature for 2 h. After completion, as determined by TLC (mobile phase: hexane:ethyl acetate:triethylamine, 15:5:3, *v/v/v*), the solvent was evaporated under reduced pressure. The crude product was dissolved in ethyl acetate (7 mL) and washed with 2% potassium carbonate (1 x 7 mL). The organic phase was then dried over anhydrous sodium sulfate and evaporated under reduced pressure. The product was purified by column chromatography (mobile phase: hexane:ethyl acetate:triethylamine, 7:2:1, *v/v*). **Yield:** 22% (15 mg) as yellowish oil. $R_f$ 0.45 (mobile phase: hexane:ethyl acetate:triethylamine, 15/5/3, *v/v*). **Characterization:** $^1$H NMR (500 MHz, $CDCl_3$) δ 8.69 (dd, *J* = 5.2, 1.7 Hz, 1H, Py), 8.56 (dd, *J* = 5.0, 1.7 Hz, 1H), 8.34 (s, 1H, CH), 7.84 – 7.78 (m, 1H, Py), 7.77 – 7.70 (m, 1H, Py), 7.68 – 7.59 (m, 2H, Py), 7.25 – 7.20 (m, 1H, Py), 7.19 – 7.15 (m, 1H, Py), 3.04 (s, 1H, CH), 2.78 (s, 3H, $CH_3$), 1.89 – 0.79 (m, 10H, *c*Hex).$^{13}$C NMR (126 MHz, $CDCl_3$) δ 161.11, 156.87, 155.71, 149.20, 149.06, 136.72, 135.96, 135.03, 126.18, 122.61, 122.43, 122.25, 62.41, 31.59, 30.90, 25.63, 25.33. **MS-ESI$^+$:** $[M+H]^+$ 322.

### *1.4. N-cyclohexyl-N-methylsemicarbazide - precursor for synthesis of DpC-S*

**Synthesis:** To a stirred solution of bis(trichloromethyl)carbonate (7.6 g, 25.6 mmol) and dry pyridine (12.4 mL, 154 mmol) in dry $CH_2Cl_2$ (50 mL), a solution of the *N*-methylcyclohexylamine (10 mL, 8.68g, 76.7 mmol) in dry $CH_2Cl_2$ (30 mL) was added. The mixture was stirred at room temperature for 2 h and washed with 2 M HCl (2 x 60 mL). The organic phase was then washed with saturated $NaHCO_3$ (2 x 60 mL) and brine (1 x 60 mL), dried over anhydrous sodium sulfate and evaporated under vacuum. Hydrazine hydrate (80%; 24 mL, 380 mmol) in ethanol (50 mL) was then added to the residue under vigorous stirring. The reaction mixture was stirred for 30 min and evaporated under vacuum. The residue was dissolved in $CHCl_3$ (200 mL) and extracted with water (3 x 100 mL). Subsequently, the organic layer was dried over anhydrous sodium sulfate, evaporated and the product was purified using column chromatography (mobile phase: chloroform:methanol 15:1, *v/v*). **Yield:** 61% (8 g) as a yellowish solid. **Characterization:** $^1$H NMR (300 MHz, DMSO) δ 7.32 (s, 1H), 3.91 – 3.74 (m, 3H), 2.59 (s, 3H), 1.76 – 1.64 (m, 2H), 1.61 – 1.17 (m, 7H), 1.11 – 0.94 (m, 1H). $^{13}$C NMR (75 MHz, $CDCl_3$) δ 160.17, 53.05, 29.99, 27.58, 25.60, 25.27.

### *1.5. Di(2-pyridyl)ketone 4-cyclohexyl-4-methylsemicarbazone (DpC-S)*

**Synthesis:** Di(2-pyridyl)ketone (0.5g, 2.7 mmol) and *N*-cyclohexyl-*N*-methylsemicarbazide (0.56 g, 3.2 mmol) were dissolved in ethanol (10 mL), 5 drops of acetic acid were added and the reaction was refluxed overnight. The reaction mixture was then evaporated and the product was isolated using column chromatography (mobile phase: ethyl acetate). The product was recrystallized from ethanol/water. **Yield**: 22% (0.2 g) as colourless crystals. **Characterization:**

$^{1}H$ NMR (300 MHz, $CDCl_3$) δ 14.41 (s, 1H, NH), 8.73 – 8.63 (m, 1H, Py), 8.61 – 8.53 (m, 1H, Py), 8.11 – 8.03 (m, 1H, Py), 7.87 – 7.76 (m, 2H, Py), 7.65 – 7.59 (m, 1H, Py), 7.38 – 7.27 (m, 2H, Py), 4.08 (s, 1H, CH), 2.94 (s, 3H, $CH_3$), 1.95 – 1.01 (m, 10H, *c*Hex). $^{13}C$ NMR (75 MHz, $CDCl_3$) δ 156.07, 154.78, 152.30, 147.23, 146.93, 140.45, 137.78, 137.09, 126.30, 124.87, 123.51, 123.16, 54.52, 30.43, 28.22, 25.80, 25.50. **MS-ESI$^+$:** $[M+H]^+$ 338.

## 2. Detailed analytical condition of LC-MS methods

### 2.1. *HPLC-MS methods to search for metabolites*

A Shimadzu Prominence HPLC system (Duisburg, Germany) consisting of a DGU-20A3 degasser, two LC-20 AD pumps, a SIL-20 AC autosampler, a CTO-20 AC column oven and a CBM 20A communication module was utilized. This was coupled with a Thermo Finnigan LCQ Advantage Max ion-trap mass spectrometer (San Jose, CA, USA) with electrospray ionization (ESI) source. The obtained data were processed with Thermo Finnigan Xcalibur software (version 2.0).

A Discovery® HS C18 (75 × 4.6 mm, 3 µm, Supelco, Germany) column with the same type of guard column were used for all studies. Studies were performed using a mobile phase consisting of 2 mM ammonium formate (A) and acetonitrile (B) in the following gradient mode (0 min – 20% B, 20 min – 60% B, 38 min – 60% B, 38.01 min – 20% B, 50 min – 20% B). The mass spectrometer was tuned automatically for Dp44mT with the following outcomes: **(I)** Positive mode: spray voltage of 3 kV, capillary voltage of 28 V, capillary temperature of 200°C, sweep and auxiliary gas flows of 45 and 20 arbitrary units, respectively. **(II)** Negative mode: an identical setting was used with the exception of the spray voltage of 4.5 kV and capillary voltage

of -10 V. The automatically tuned values for DpC were very similar to those for Dp44mT, and thus, the set-up was kept the same for both analytes. In order to detect any possible metabolites, full scans (*m/z* 100-1000) in both positive and negative modes as well as selected ion monitoring (SIM) for the metabolites proposed previously (Stariat, J. *et al.* Anal Bioanal Chem. 2013;405(5):1651-1661) were implemented.

## ***2.2. UHPLC-MS/MS methods for quantitative assay of the drugs and metabolites used in the pharmacokinetic study***

The mobile phase consisted of 2 mmol·L$^{-1}$ ammonium formate in aqueous solution with the addition of $K_2EDTA$ (5 µmol·L$^{-1}$, component A) and acetonitrile (component B) was used in the following gradient modes:

| **Dp44mT** | | **DpC** | |
|---|---|---|---|
| time (min) | B (%) | time (min) | B (%) |
| 0.00 | 10 | 0.00 | 30 |
| 1.00 | 10 | 2.00 | 30 |
| 3.50 | 50 | 5.50 | 70 |
| 4.00 | 50 | 6.00 | 70 |
| 4.01 | 10 | 6.01 | 30 |
| 6.00 | 10 | 8.00 | 30 |

The LCMS-8030 triple quadrupole mass detector was set-up as follows: Interface voltage 4.5kV, DL temperature 250°C, heat block temperature 400°C, nebulizing gas flow rate 3 L/min, drying gas flow rate 15 L/min, CID gas pressure 230 kPa, dwell time either 10 ms (Dp44mT, Dp4mT and IS) or 20 ms (DpC, DpC-A). Quantitation was performed in selected reaction monitoring mode (SRM) using the precursor and product ions specified below:

| **Compound** | **1st SRM** | **CE (eV)** | **2nd SRM** | **CE (eV)** | **3rd SRM** | **CE (eV)** |
|---|---|---|---|---|---|---|
| Dp44mT | 285.90 → 241.10 | 12.0 | 285.90 → 79.00 | -31.0 | 285.90 → 183.20 | -19.0 |
| Dp4mT | 272.10 → 147.90 | 14.0 | 272.10 → 240.95 | -11.0 | 272.10 → 183.15 | -18.0 |
| DpC | 353.90 → 240.75 | 14.0 | 353.90 → 79.15 | -37.0 | 353.90 → 183.15 | -24.0 |
| DpC-A | 322.00 → 78.25 | 42.0 | 321.70 → 160.75 | -29.0 | 322.00 → 55.20 | -48.0 |
| IS | 268.90 → 223.95 | 12.0 | 268.90 → 181.00 | -24.0 | 268.90 → 77.00 | -39.0 |

The UHPLC-MS/MS methods for quantitative assays were validated with respect to selectivity, linearity, precision, accuracy, stability, recovery, matrix effects and dilution integrity (see Supplementary Table 1-3). Selectivity was assessed using blank plasma from eight individual sources. Linearity was examined over the concentration ranges described in Supplementary Table 1 using a weighted ($1/x^2$) linear regression in GraphPad Prism 6 (v. 6.04, GraphPad Software Inc., U.S.A.).

Precision and accuracy of the method were investigated at three concentration levels (low, medium and high) (see Supplementary Table 1) in replicate ($n$ = 5) on one day for intra-day precision and accuracy. The same experiment was repeated on a different day for inter-day evaluation. Stability was assessed at low, medium and high concentration levels. Long-term stability was assessed for plasma samples at -80°C over a period of 7 days (three aliquots per level; Supplementary Table 3). Post-preparative stability was checked in the autosampler (10°C) for 24 h (five aliquots per level; Supplementary Table 3). Extraction recovery was expressed as

ratio of the peak areas of analytes in extracted "spiked" samples and the peak areas of analytes in blanks "spiked" after extraction (Supplementary Table 2).

Matrix effects were tested by comparison of the peak area of the analyte in post-extracted plasma to the peak area of a standard of the same concentration dissolved in 50% acetonitrile. Matrix effects were expressed as both absolute matrix factor (MF) and IS-normalized matrix factor. Both recovery and matrix effects were evaluated at low, medium and high concentrations for the analytes and at one concentration of IS for each analyte (0.25 $\mu mol \cdot L^{-1}$ for Dp44mT and 1 $\mu mol \cdot L^{-1}$ for DpC). For results see Supplementary Table 2.

Dilution integrity was tested by analysis of plasma samples "spiked" at either 7 $\mu mol \cdot L^{-1}$ of Dp44mT and Dp4mT or 12 $\mu mol \cdot L^{-1}$ of DpC and DpC-A, which were diluted 10 times with pooled blank plasma. Dilution integrity was expressed as precision and accuracy of the concentration determined by comparison to the concentration added (see Supplementary Table 3).

## SUPPLEMENTAL FIGURES

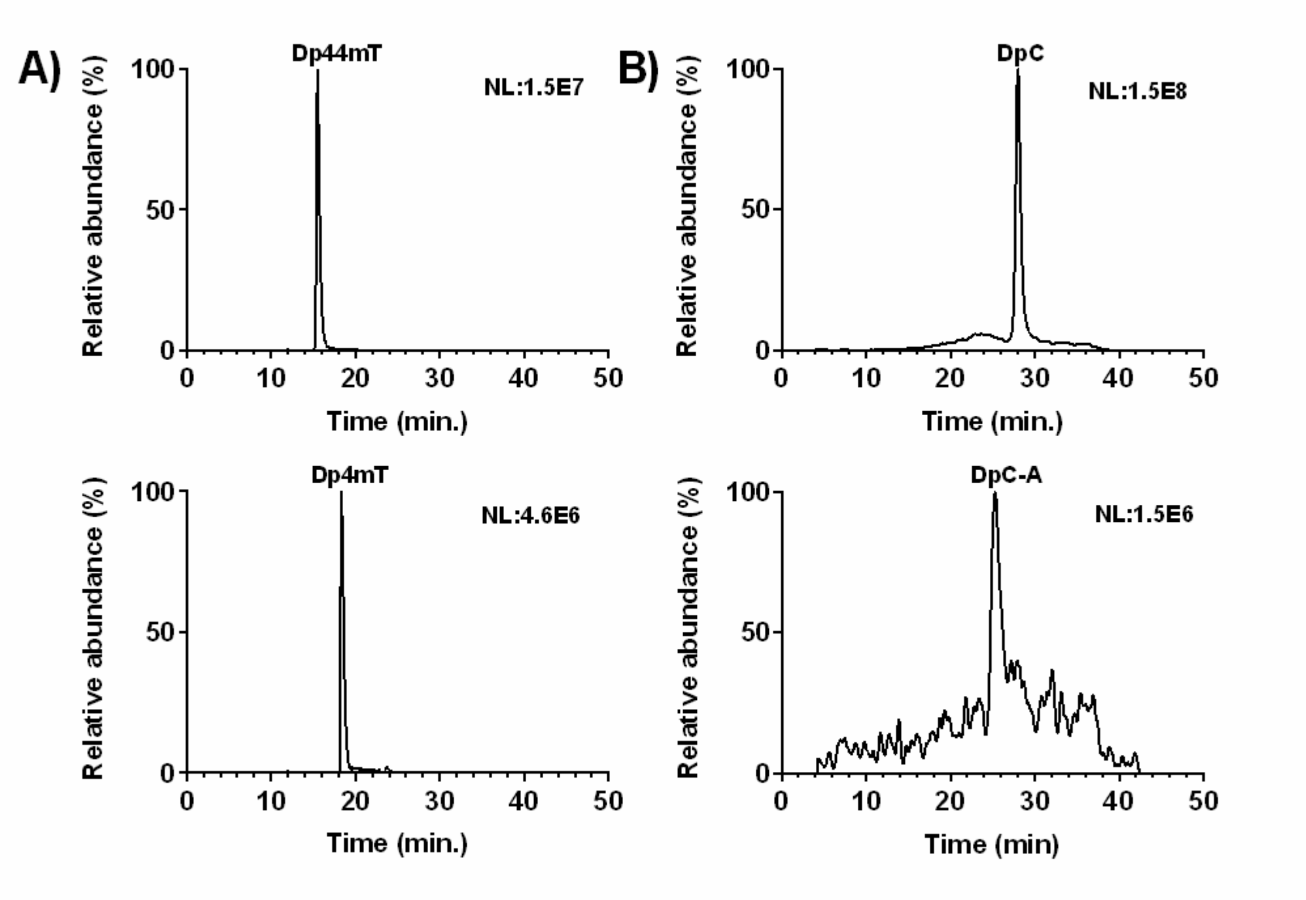


**Supplemental Fig. 1. Chromatogram of HPLC-MS analysis of plasma taken after *i.v.* administration of: (A) Dp44mT or (B) DpC to rats.** The chromatograms are recorded in selected ion monitoring and the intensity of signal is presented as normalization level values (NL).

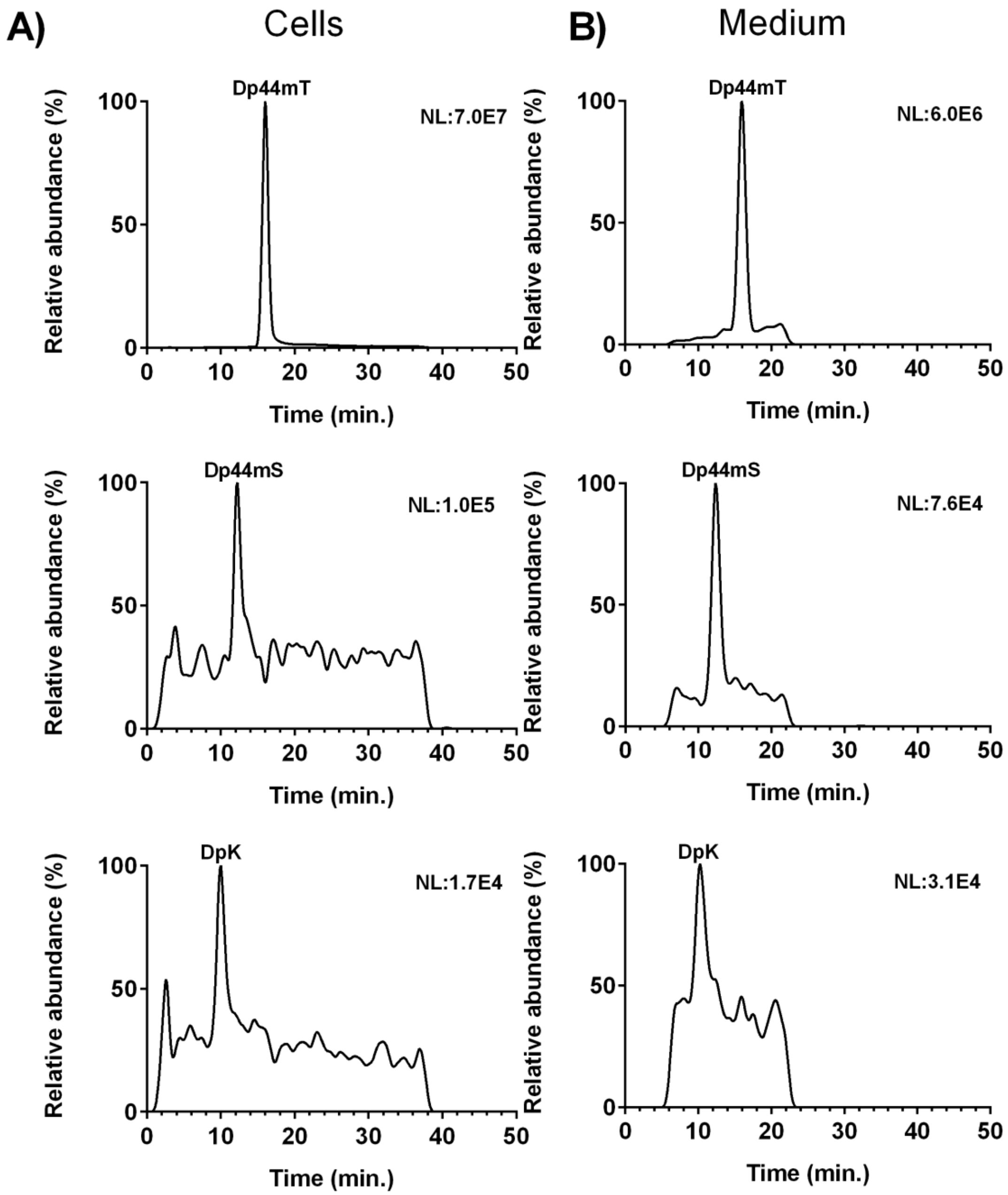


**Supplemental Fig. 2. Chromatogram of HPLC-MS analysis of: (A) MCF-7 cells; and (B) the corresponding media obtained from incubation with Dp44mT.** The chromatograms are recorded in selected ion monitoring and the intensity of signal is presented as normalization level values (NL).

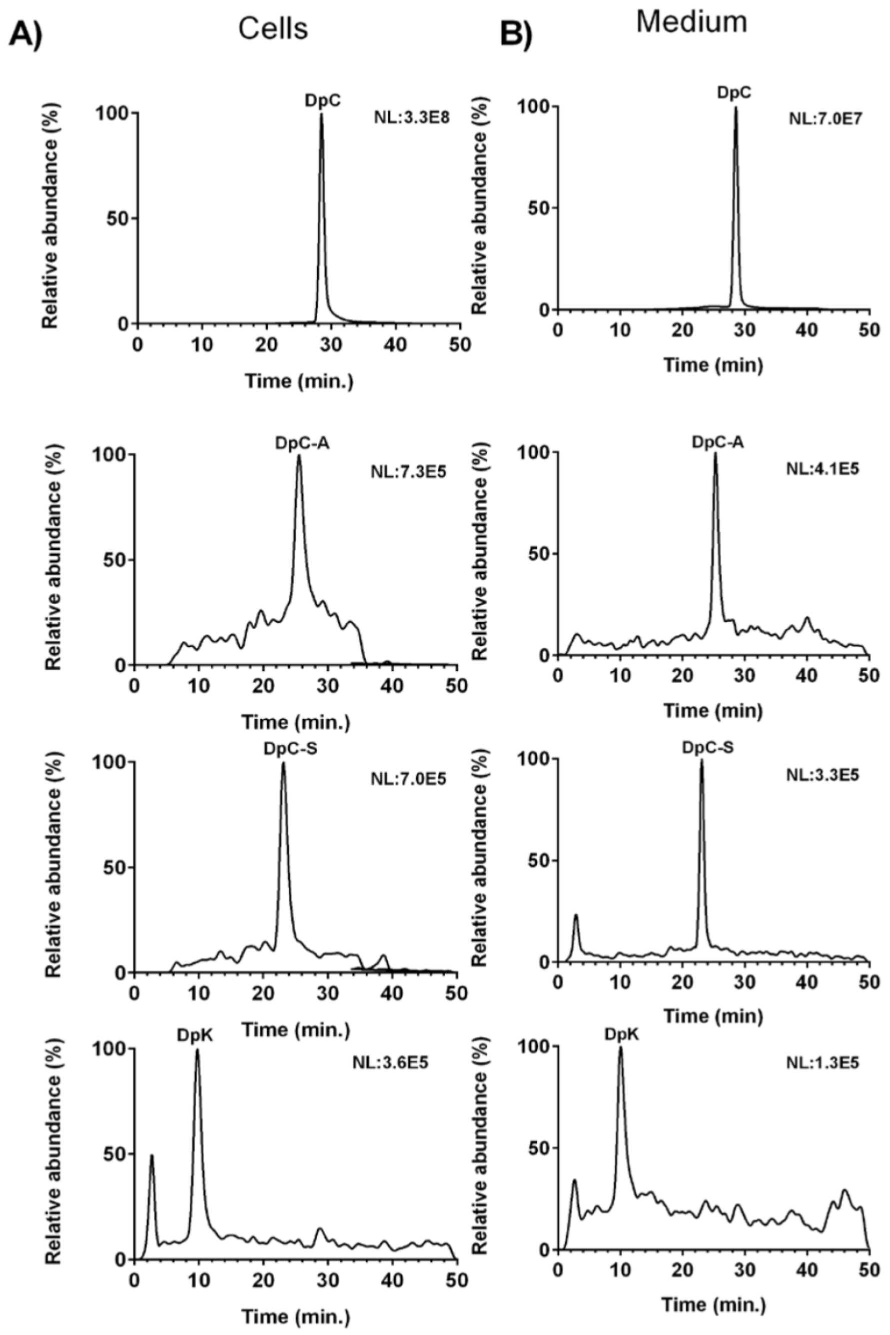


**Supplemental Fig. 3. Chromatogram of HPLC-MS analysis of: (A) MCF-7 cells; and (B) the corresponding media obtained from incubation with DpC.** The chromatograms are recorded in selected ion monitoring and the intensity of signal is presented as normalization level values (NL).

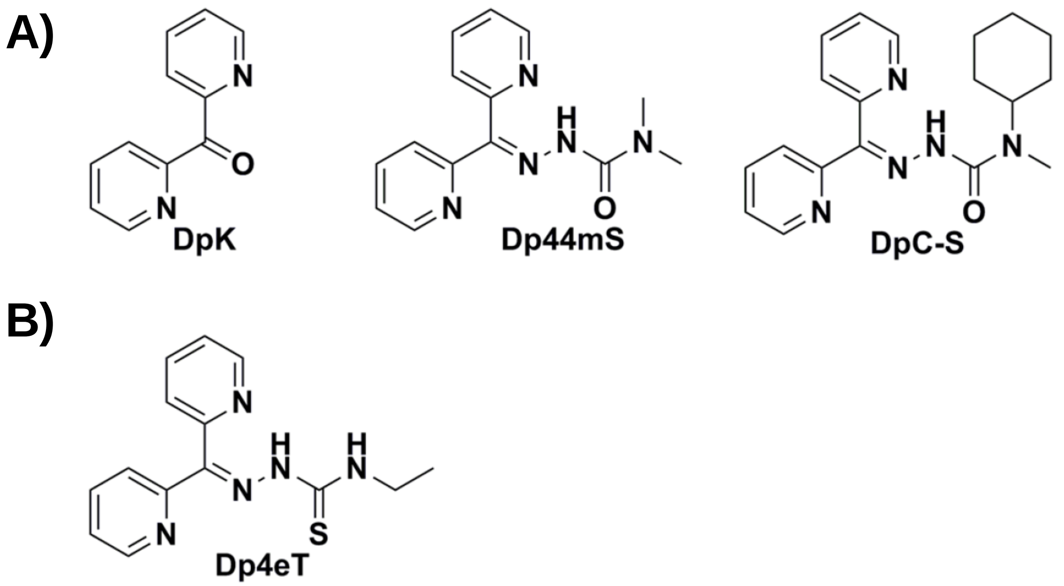


**Supplemental Fig. 4. Chemical structures of: (A) the predicted respective metabolites of Dp44mT and DpC (di(2-pyridyl)ketone [DpK]; di(2-pyridyl)ketone 4,4-dimethylsemicarbazone [Dp44mS]; and di(2-pyridyl)ketone 4-cyclohexyl-4-methylsemicarbazone [DpC-S]); and (B) the adjuvant chelator (di(2-pyridyl)ketone 4-ethyl-3-thiosemicarbazone; Dp4eT) used to improve extraction.**

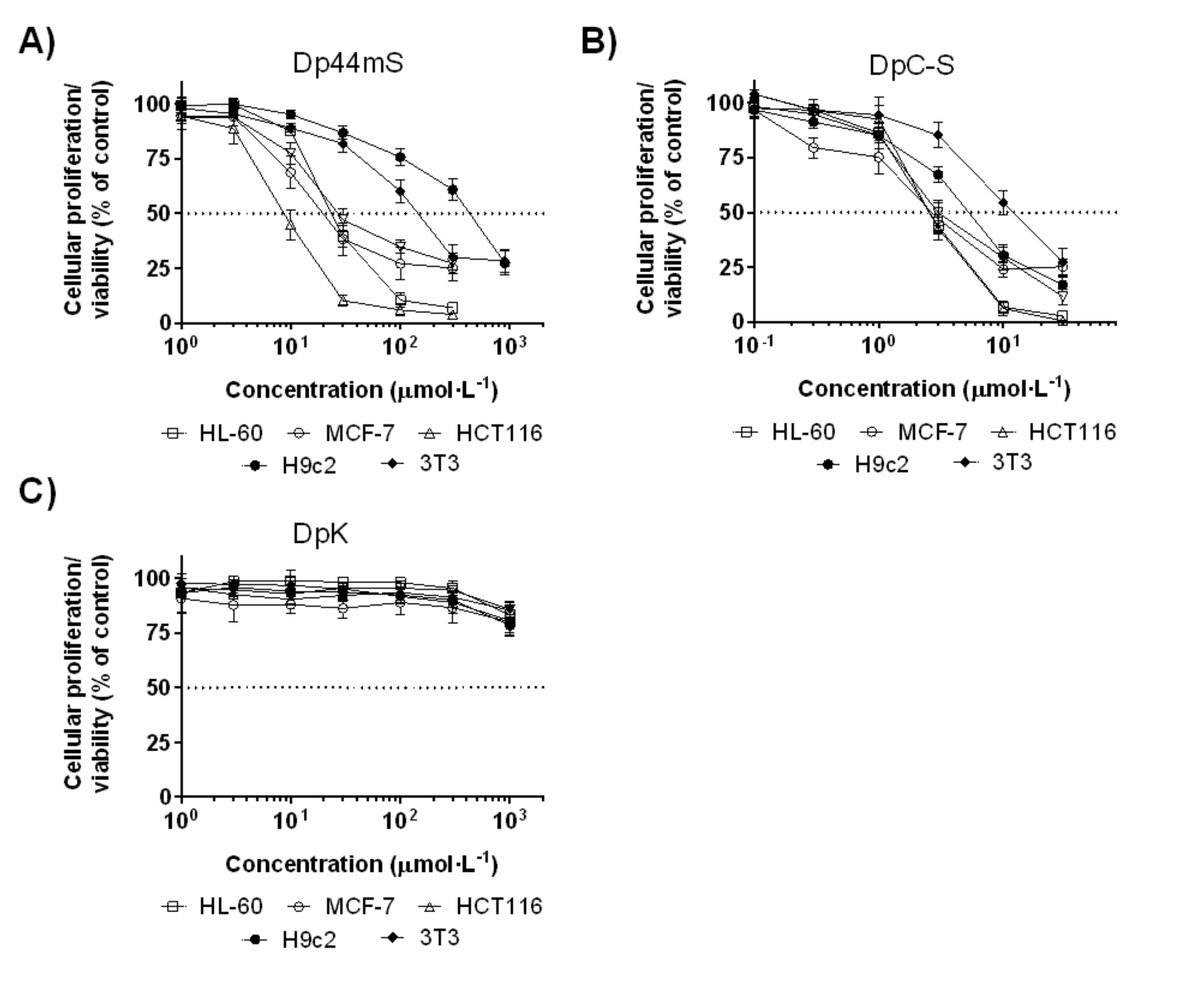


**Supplemental Fig. 5. Cytotoxic effects of the putative respective metabolites of the parent thiosemicarbazones, Dp44mT and DpC: (A)** Dp44mS; **(B)** DpC-S; and (**C)** DpK. The anti-proliferative/cytotoxic effects of the compounds were examined at different concentrations after a 72 h/37$^{o}$C incubation with cancer (HL-60, MCF-7 and HCT116) and non-cancer (H9c2 and 3T3) cells lines. Proliferation/viability was determined using the MTT assay. Data are mean ± SD ($n \geq 4$).

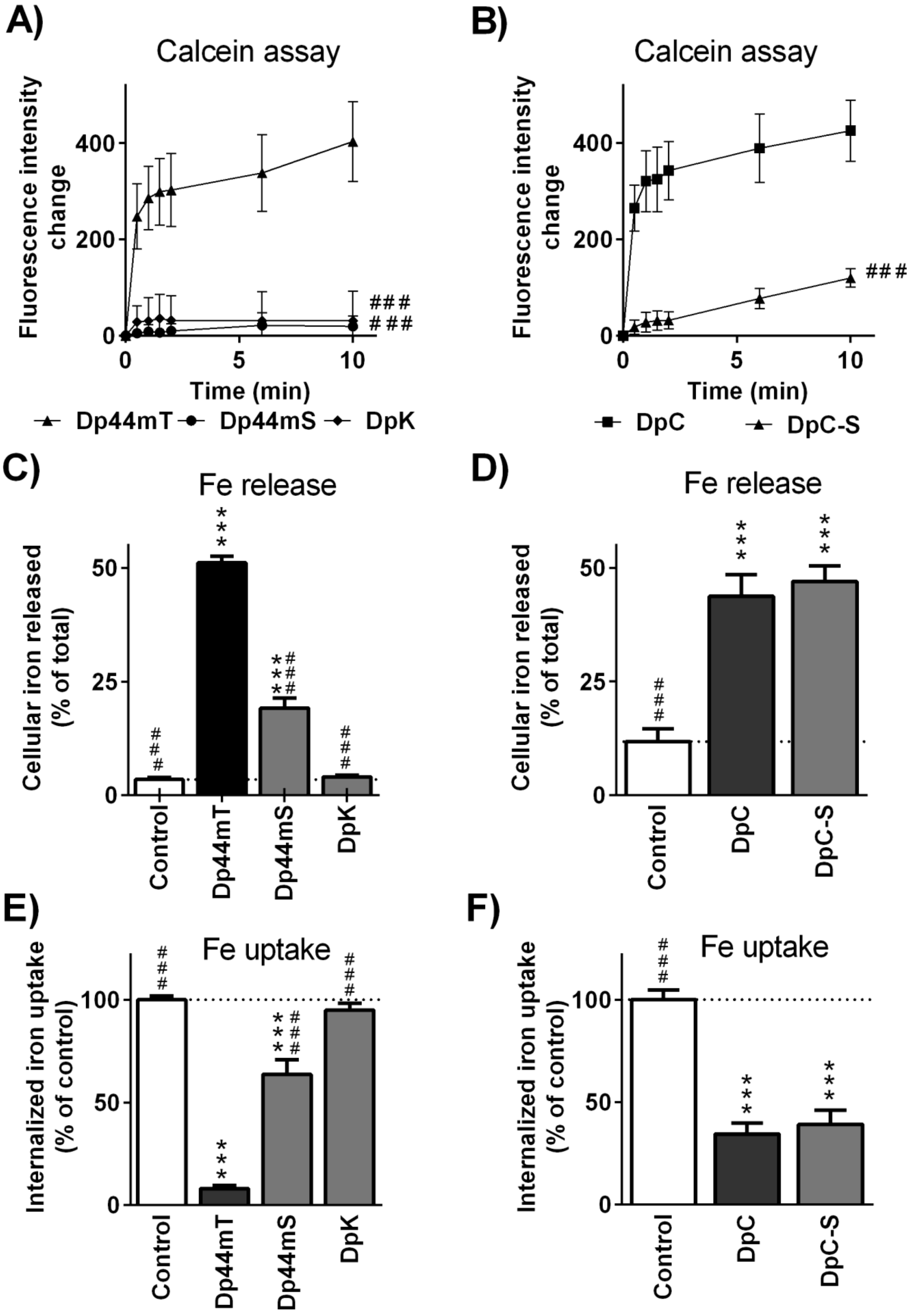
A)
Calcein assay
Fluorescence intensity change
Time (min)
Dp44mT
Dp44mS
DpK
B)
Calcein assay
Fluorescence intensity change
Time (min)
DpC
DpC-S
C)
Fe release
Cellular iron released (% of total)
Control
Dp44mT
Dp44mS
DpK
D)
Fe release
Cellular iron released (% of total)
Control
DpC
DpC-S
E)
Fe uptake
Internalized iron uptake (% of control)
Control
Dp44mT
Dp44mS
DpK
F)
Fe uptake
Internalized iron uptake (% of control)
Control
DpC
DpC-S

**Supplemental Fig. 6. Ability of (A) Dp44mT and its putative metabolites, Dp44mS and DpK; and (B) DpC and its putative metabolite, DpC-S, to bind iron from the intracellular labile iron pool (LIP) determined by the fluorimetric calcein-AM assay. (C, D) The ability of these agents to mobilize $^{59}$Fe from prelabeled MCF-7 cells; and (E, F) to inhibit internalized $^{59}$Fe uptake from $^{59}$Fe-transferrin by MCF-7 cells.** Statistical significance (ANOVA): # $p < 0.05$, ## $p < 0.01$, ### $p < 0.001$ compared to the parent thiosemicarbazones, Dp44mT or DpC, respectively. * $p < 0.05$, ** $p < 0.01$, *** $p < 0.001$ compared to the control (untreated) group. Data are mean ± SD ($n \geq 4$).

## SUPPLEMENTARY TABLES

**Supplementary Table 1. Results of validation of the UHPLC-MS/MS methods for the quantitative assay used in the PK study**: Linearity, precision and accuracy of the assay for Dp44mT, its metabolite, Dp4mT, and DpC and its metabolite, DpC-A, in plasma. Data are presented as mean± SD ($n \geq 5$).

| **Linearity** | Slope | Intercept | $r^2$ | Range (μmol·L$^{-1}$) |
|---|---|---|---|---|
| Dp44mT | 0.00326 | -0.01068 | 0.9982 | 0.035 - 2.100 |
| Dp4mT | 0.00354 | 0.00700 | 0.9929 | 0.035 - 2.200 |
| DpC | 0.00257 | -0.05458 | 0.9739 | 0.150 - 3.000 |
| DpC-A | 0.00182 | 0.01384 | 0.9920 | 0.150 - 3.100 |

| | **Intra-day (*n* = 5)** | | | **Inter-day (*n* = 5)** | | |
|---|---|---|---|---|---|---|
| | **Precision** | | **Accuracy** | **Precision** | | **Accuracy** |
| Added (μmol·L$^{-1}$) | Found ± SD (μmol·L$^{-1}$) | RSD (%) | (%) | Found ± SD (μmol·L$^{-1}$) | RSD (%) | (%) |
| **Dp44mT** | | | | | | |
| 2.105 | 2.016 ± 0.102 | 5.06 | 95.77 | 1.958 ± 0.209 | 10.67 | 93.02 |
| 0.702 | 0.738 ± 0.085 | 11.52 | 105.13 | 0.738 ± 0.081 | 10.97 | 105.13 |
| 0.035 | 0.035 ± 0.001 | 2.86 | 100.00 | 0.035 ± 0.002 | 5.71 | 100.00 |
| **Dp4mT** | | | | | | |
| 2.187 | 1.920 ± 0.094 | 4.90 | 87.79 | 1.947 ± 0.125 | 6.42 | 89.03 |
| 0.729 | 0.739 ± 0.053 | 7.17 | 101.37 | 0.788 ± 0.096 | 12.18 | 108.09 |
| 0.036 | 0.035 ± 0.002 | 5.71 | 97.22 | 0.035 ± 0.004 | 11.43 | 100.00 |
| **DpC** | | | | | | |
| 2.969 | 3.064 ± 0.208 | 6.79 | 103.20 | 3.176 ± 0.250 | 7.87 | 106.97 |
| 1.484 | 1.503 ± 0.219 | 14.57 | 101.28 | 1.471 ± 0.207 | 14.07 | 99.12 |
| 0.148 | 0.154 ± 0.014 | 9.09 | 104.05 | 0.152 ± 0.017 | 11.18 | 102.70 |
| **DpC-A** | | | | | | |
| 3.115 | 3.156 ± 0.145 | 4.59 | 101.32 | 3.148 ± 0.179 | 5.69 | 101.06 |
| 1.558 | 1.528 ± 0.148 | 9.69 | 98.07 | 1.480 ± 0.165 | 11.15 | 94.99 |
| 0.156 | 0.153 ± 0.018 | 11.76 | 98.08 | 0.157 ± 0.019 | 12.10 | 100.64 |

**Supplementary Table 2. Validation of the UHPLC-MS/MS methods for the quantitative assay used in the PK study**. Extraction recovery and matrix factor (MF) of the assay for Dp44mT, its metabolite, Dp4mT, and DpC and its metabolite, DpC-A, in plasma. Data are presented as the mean (*n* =3).

| Concentration level (μmol·L$^{-1}$) | Extraction recovery (%) | Absolute MF (%) | IS-normalized MF (%) |
|---|---|---|---|
| **Dp44mT** | | | |
| 2.105 | 64.10 | 91.55 | 100.56 |
| 0.702 | 71.38 | 93.05 | 93.82 |
| 0.035 | 70.28 | 92.89 | 94.27 |
| **Dp4mT** | | | |
| 2.187 | 99.85 | 94.82 | 103.96 |
| 0.729 | 93.76 | 100.72 | 106.94 |
| 0.036 | 104.35 | 98.62 | 100.96 |
| **DpC** | | | |
| 2.969 | 88.74 | 103.58 | 100.36 |
| 1.484 | 63.73 | 104.28 | 104.75 |
| 0.148 | 61.24 | 99.98 | 105.40 |
| **DpC-A** | | | |
| 3.115 | 111.68 | 105.31 | 100.79 |
| 1.558 | 93.70 | 100.29 | 95.52 |
| 0.156 | 119.53 | 104.36 | 109.15 |
| **I.S.** | | | |
| 1.000 | 87.28 | 101.34 | - |
| 0.250 | 87.10 | 104.05 | - |

**Supplementary Table 3. Results of validation of the UHPLC-MS/MS methods for the quantitative assay used in the PK study.** Stability and dilution integrity of the assay for Dp44mT, its metabolite, Dp4mT, and DpC and its metabolite, DpC-A, in plasma. Data are presented as the mean ($n \geq 3$).

| Stability | | | | | |
|---|---|---|---|---|---|
| Concentration level ($\mu mol \cdot L^{-1}$) | Post-preparative (%) | Long-term (%) | Concentration level ($\mu mol \cdot L^{-1}$) | Post-preparative (%) | Long-term (%) |
| **Dp44mT** | | | **DpC** | | |
| 2.105 | 98.90 | 94.93 | 2.969 | 107.34 | 112.39 |
| 0.702 | 95.92 | 108.06 | 1.484 | 95.68 | 87.76 |
| 0.035 | 93.70 | 112.56 | 0.148 | 97.48 | 85.12 |
| **Dp4mT** | | | **DpC-A** | | |
| 2.187 | 95.44 | 90.82 | 3.115 | 99.55 | 104.23 |
| 0.729 | 111.72 | 90.83 | 1.558 | 93.89 | 105.54 |
| 0.036 | 100.24 | 94.78 | 0.156 | 101.11 | 87.83 |

| Dilution integrity | | | |
|---|---|---|---|
| Spiked ($\mu mol \cdot L^{-1}$) | Determined ± SD ($\mu mol \cdot L^{-1}$) | RSD (%) | Accuracy (%) |
| **Dp44mT** | | | |
| 7.03 | 7.76 ± 0.76 | 9.79 | 110.38 |
| **Dp4mT** | | | |
| 7.30 | 6.68 ± 0.74 | 11.08 | 91.51 |
| **DpC** | | | |
| 11.88 | 10.89 ± 1.32 | 12.12 | 91.67 |
| **DpC-A** | | | |
| 12.46 | 11.68 ± 0.49 | 4.20 | 93.74 |

**Supplementary Table 4**: **Cytotoxic effects of the respective putative metabolites of Dp44mT and DpC (Dp44mS, DpK and DpC-S) towards both cancer (HL-60, MCF-7 and HCT116) and non-cancer cell lines (H9c2 and 3T3).** The compounds were incubated with cells for 72 h/37°C. Cell proliferation/viability was determined using the MTT assay and the $IC_{50}$ values (half-maximal inhibitory concentrations) were calculated using CalcuSyn 2.0 software. Data are mean ± SD ($n \geq 4$ experiments).

| | $IC_{50}$ (μmol·L$^{-1}$) | | | | |
|---|---|---|---|---|---|
| | **HL-60** | **MCF-7** | **HCT116** | **H9c2** | **3T3** |
| **Dp44mS** | 27.32 ± 3.56 | 29.38 ± 8.63 | 9.31 ± 1.58 | 366.93 ± 63.35 | 138.79 ± 28.87 |
| **DpK** | >1000 | >1000 | >1000 | >1000 | >1000 |
| **DpC-S** | 2.57 ± 0.33 | 3.00 ± 0.81 | 3.03 ± 0.91 | 5.59 ± 0.82 | 12.83 ± 2.87 |
